# Efficient estimation of proton exchange membrane fuel cells parameters using a hybrid swarm intelligent algorithm

**DOI:** 10.1038/s41598-025-14297-1

**Published:** 2026-01-08

**Authors:** Pankaj Sharma, Rohit Salgotra, Saravanakumar Raju, Szymon Łukasik, Amir H. Gandomi

**Affiliations:** 1 School of Electrical Engineering, Vellore Institute of Technology, Vellore, India; 2 Electrical and Electronics Engineering, SR University, Warangal, Telangana, India; 3 Faculty of Physics and Applied Computer Science, AGH University of Kraków, Kraków, Poland; 4 Faculty of Engineering and IT, University of Technology Sydney, Sydney, Australia; 5 Center of Excellence in Artificial Intelligence, AGH University of Kraków, Kraków, Poland; 6 University Research and Innovation Center (EKIK), Óbuda University, Budapest, Hungary; 7 Department of Computer Science, Khazar University, Baku, Azerbaijan

**Keywords:** Evolutionary algorithms, PEMFCs, Parameter identification, GPC algorithm, Polarization curves, Engineering, Computational science

## Abstract

The identification of unknown parameters for proton exchange memberane fuel cells (PEMFCs) using nature-inspired optimization algorithms has emerged as a significant field of research in recent years. In the present study, a novel approach is presented, namely the hybrid Gray Particle Cuckoo (GPC) algorithm based on the hybrid properties of the grey wolf optimizer (GWO), particle swarm optimization (PSO), and cuckoo search (CS) to address the identification problem associated with PEMFCs. The effectiveness of the proposed GPC algorithm is evaluated on four commercially available PEMFCs (BCS500-W, Ballard Mark V, Temasek, as well as NedStack PS6). The fitness function has been expressed as the sum of the squared errors (SSE) that occurred between the estimated voltage and the data that corresponded to it. To further validate the model of the PEMFC, it is contrasted with other complex algorithms. The GPC algorithm showed the lowest SSE across all cases, resulting in SSE values of 0.011699, 0.813912, 2.267687, and 0.123276775 for the BCS500-W, Ballard Mark V, NedStack PS6 and Temasek PEMFC stack, respectively. Also, the PEMFC stacks are evaluated using different partial temperature and pressure conditions. In addition to real-world challenges, the GPC algorithm has been assessed on 100-digit CEC 2019 benchmarks and contrasted to other MH algorithms. Furthermore, both the parametric and non-parametric statistical tests are conducted to evaluate the efficacy of the GPC algorithm. The results in terms of mean square error (MSE), individual absolute error (IAE), mean bias error (MBE), mean absolute error (MAE), and root-mean-square error (RMSE) demonstrate that the GPC algorithm is the optimal choice contrasted to other algorithms due to its better solution quality and faster convergence time.

## Introduction

The depletion of fossil fuels, caused by the increasing consumption of energy, as well as increased awareness of environmental conservation, has led individuals and governments to focus on alternative energy sources. As a result, researchers have presented significant interest in exploring other energy sources that are more environmentally friendly, such as wind, solar and wave energy^1,2^. These sources have gained considerable attention because of their potential to mitigate the negative impacts of traditional energy production methods on the environment. Therefore, various studies and investigations have been conducted to assess the feasibility and efficiency of these greener energy sources. The main obstacles associated with the sources mentioned are their unpredictable characteristics and dependence on climatic factors. However, these constraints have clearly highlighted the crucial need for energy storage. Hydrogen, a topic of current interest, has the potential to serve as an energy storage medium to effectively store renewable energy until it can be converted to electricity by an energy conversion device^3^. A fuel cell (FC) is a very important technology for converting energy, usually generating electricity, by employing a chemical reaction between hydrogen as well as oxygen. The PEMFC has become popular in many fields, including automotive, on-site generation, as well as portable electronic devices, because of its advantages, which include high power density, low operating temperature, and solid electrolyte^4^.

Enhancing the efficiency as well as performance of PEMFCs has become an important area of study. The mechanical model incorporates the internal dynamics of cells through mass and heat conservation laws, alongside chemical reaction equations, whereas the empirical model represents the external properties of cells using empirical formulas derived from experimentation, which are less complex than those of the mechanical model. The precise identification of the model’s parameters remains to be a considerable challenge^5,6^. This paper utilises a semi-empirical model that integrates a mechanism model with empirical components, presenting a voltage model that thoroughly addresses active polarisation loss, ohmic polarisation loss, and concentration polarisation loss^7,8^. A precise mathematical model is essential to accurately represent the actual behavior of the system under various operating scenarios^9,10^. Identifying the optimal values of the unknown parameters results in a mathematical model that exhibits a high level of precision^11^. As a result of the related non-linearity of the FC, the modeling procedure becomes complex due to the presence of certain unknown parameters in the manufacturer’s datasheet^12^.

In recent decades, extensive research has been done on estimating unknown parameters in the literature, categorising studies into two primary areas, such as MH as well as deterministic optimization techniques. Deterministic optimization methods, including derivative-based optimization as well as linear programming, depend on precise mathematical principles. MH and deterministic methods offer unique advantages that depend on the specific characteristics of the problem at hand^13,14^. Deterministic techniques provide efficient and accurate solutions for small-scale and linear problems, ensuring optimal results. These methods may be insufficient for complex problems characterised by multiple variables and non-linear relationships, as they are prone to convergence on suboptimal solutions. The MH optimization algorithms have gained significant popularity for addressing various optimisation problems due to their flexibility, derivation-free approach, and simplicity^15–17^. The complicated nature of the PEMFC parameter identification challenge has resulted in the inadequate performance of conventional search strategies in accurately determining the best possible solutions^18,19^. In addition, various other MHA have been implemented to improve both the accuracy and the effectiveness of the model, including Dimension Learning-based Modified Grey Wolf Optimizer (DLHMGWO)^5^, Improved Heap-based optimizer (IHBO)^20^, Lightning Search Algorithm (LSA)^21^, Artificial Hummingbird Algorithm (AHA)^22^, Repairable Grey Wolf Optimization (RGWO)^23^, Shark Smell Optimizer (ShSO)^24^, Manta Rays Foraging Optimizer (MRFO)^25^, Flower Grey INFO Naked (FGIN)^26^, Hybrid Vortex Search Differential Evolution (VSDE)^27^, Honey Badger Optimization Algorithm (HBA)^28^, Improved Artificial Bee Colony (IABC)^29^, Imperialist Competitive Algorithm (ICA)^30^, Flower Pollination Algorithm (FPA)^31^, Pathfinder Algorithm (PFA)^32^, Transient Search Optimization (TSO)^33^, Dandelion Optimize (DO)^34^, Hunger Games Search Marine Predator Algorithm (HGS-MPA)^35^, Aquila Optimizer Arithmetic Algorithm Optimization (AOAAO)^36^, Parrot Optimizer (PO)^37^, Hybrid Artificial Bee Colony Differential Evolution Optimizer (ABC-DE)^38^, Chaotically based-bonobo optimizer (CBO)^39^, Enhanced Salp Swarm Algorithm (ESSA)^40^, Honey Badger Optimizer (HBO)^9^, Autonomous Groups Particle Swarm Optimization (AGPSO)^8^, Gorilla Troops Optimizer (GTO)^41^, Enhanced Walrus Optimization (EWO)^42^, Improved Artificial Ecosystem Optimizer (IAEO)^43^, Combined Owl Search Algorithm (COSA)^44^, War Strategy Optimization (WSO)^45^, Improved Fish Migration Optimizer (IFMO)^46^, Ali Baba and forty thieves (ABFT)^47^, Puma Optimizer (PuO)^48^, Kepler Red Meerkat Grey (KRMG)^49^ and so on. Also, a comparative literature table has been included in order to clarify existing research on PEMFC parameter estimation, summarizing essential elements such as optimization algorithm, PEMFC stack utilization, objective functions, and the use of statistical analysis is given in Table 1.

**Table 1:** Overview of existing works on PEMFC parameter estimation.

S.No	Optimization	Year	PEMFC stack	Objective functions	Other analysis	Reference
1	DLHMGWO	2025	Heliocentris FC50, BCS- 500 W 250 W, AVISTA SR-12 500 W, Temasek 1 kW	SSE	RMSE, boxplots, convergence curve	^5^
2	HGS-MPA	2025	50-W stack, NedStack PS6, BCS 500-W	SSE	Convergence curve	^35^
3	HGS-MPA	2025	50-W stack, NedStack PS6, BCS 500-W	SSE	Convergence curve	^35^
4	AOAAO	2025	Horizon 500 W, BCS 500 W, Nedstack PS6, H-12 , 500 W SR-12	SSE	MBE, Box-Plot, Convergence Curve	^36^
5	PO	2025	STD 250 W, BCS 500 W, SR-12 W, Horizon H-12, Ballard Mark V, Nedstack 600 W PS6,	SSE	Box-Plot, Convergence Curve	^37^
6	ABFT	2025	Ballard Mark V, NedStack PS6, 250 W Stack, BCS 500 W	SSE	Friedman’s, Wilcoxon rank test, convergence, box plot	^47^
7	PuO	2025	NedStack PS6, Avista SR-12, Horizon H-12, BCS 500 W, 250 W, Ballard Mark V	SSE	Convergence, box plot	^48^
8	FGIN	2024	Stack 250 W, Ballard Mark V, Horizon H-12, NedStack PS6, BCS 500-W	SSE	IAE, MAE, MAPE, MBE, MSE, RMSE, Convergence curve	^26^
9	ESSA	2024	250 W, BCS500	SSE	RMSE, MAE, RE, convergence	^40^
10	AGPSO	2024	250Wstack and BCS-500W	TSEs	RMSE, MAE, RE, convergence, box plot	^8^
11	EWO	2024	AVISTA SR-12 500 W, TEMASEK 1 kW, Nedstack PS, 250 W	SSE	Convergence	^42^
12	WSO	2024	Horizon 500 W, NedStackPS6, BCS500W, 250 W	SSE	Convergence	^45^
13	KRMG	2024	Horizon H 12, Ballard Mark-V, Stack 250 W	SSE	RMSE, MAE, IAE, MBE, MSE, MAPE, Friedman’s, Wilcoxon rank test	^49^
14	AHA	2023	250 W, BCS 500 W, NedStack PS6, H-12, Mark V5, SR-12	SSE	Convergence curve, Boxplot	^22^
15	RGWO	2023	250 W	SSE	-	^23^
16	IABC	2023	NedStack PS6 stack, 250W FC, BCS 500W	SSE	Convergence curve, ANOVA	^29^
17	CBO	2023	250 W, 500 W stacks	SSE	Friedman rank, mean convergence	^39^
18	DO	2023	Ballard Mark V and BCS 500-W	SSE	Convergence curve	^34^
19	GTO	2023	BCS 500-W, Nedstack PS6, 250-W	SSE	Convergence	^41^
20	LSA	2022	BCS 500 W, Nedstack PS6 6 kW, Ballard Mark V 5 kW	SSE	Convergence curve	^21^
21	HBA	2022	250-W stack, NedStack PS6, BCS 500-W	SSE	Convergence curve	^28^
22	TSA	2022	Ballard, Mark V, Nedstack PS6, Horizon H-12 stacks	SSE	Convergence curve	^33^
23	ABC-DE	2022	NedStack PS6, Modular SR-12, Ballard Mark V, Horizon H-12	SSE	MAE, RMSE, convergence	^38^
24	HBO	2022	Ballard Mark, SR-12, 250 W stacks	SQEs	Convergence	^9^
25	PFA	2021	Mark V, H-12	SSE	Convergence curve	^32^
26	IHBO	2021	500 W BCS, AVISTA SR-12 500 W, NetStack, H-12 stack	SSE	MAE, MAPE, convergence curve	^20^
27	IAEO	2021	BCS 500-W, NedStack PS6, 250 W stack	SSE	Wilcoxon test, convergence, box plot	^43^
28	VSDE	2020	250 W stack, BCS 500-W, SR-12 500 W, NedStack PS6	SSE	Convergence curve	^27^

Now, there is the shortage of a dependable and efficient method for getting an accurate estimating procedure that can serve as a unique reference for research objectives. In addition, the no-free lunch (NLF) theorem motivates researchers to create novel optimization techniques or enhance/hybrid existing ones to address the real-world challenges across several domains^50^. Thus, a research gap needs to be addressed by determining the most appropriate algorithm for PEMFC and exploring other algorithms that have not been utilized in the PEMFC area. These MHAs provide several approaches to optimize the parameters of PEMFC systems and enhance their performance. However, it is possible for them to get trapped in local minima while performing the search, resulting in a gradual decline in their efficiency with every repetition. This paper presents a novel hybrid optimization (GPC) algorithm to optimally estimate the unknown parameters of PEMFC. Furthermore, Eleven MH optimization algorithms consisting of the Zebra Optimization Algorithm (ZOA)^51^, sinh cosh optimizer (SCHO)^52^, Propagation Search Algorithm (PSA)^53^, SABO^54^, Young’s Double-Slit Experiment (YDSE)^55^, exponential distribution optimizer (EDO)^56^, RIME^57^, Chernobyl Disaster Optimizer (CDO)^58^, Coati Optimization Algorithm (COA)^59^, Harris Hawks Optimizer (HHO)^60^, and GWO^61,62^, and the results obtained through these eleven algorithms are also compared with GPC algorithm. To further validate the GPC algorithm, it has been tested on CEC 2019 benchmark challenges^63,64^ and compared to some of the well-known and recently presented algorithms including, BWOA^65^, CDO^58^, COA^59^, flower pollination algorithm (FPA)^66^, HHO^60^, YDSE^55^, ZOA^51^, ARNMRA^67^, FROBLGJO^68^, as well as jDE100^69^.

**Fig. 1:**
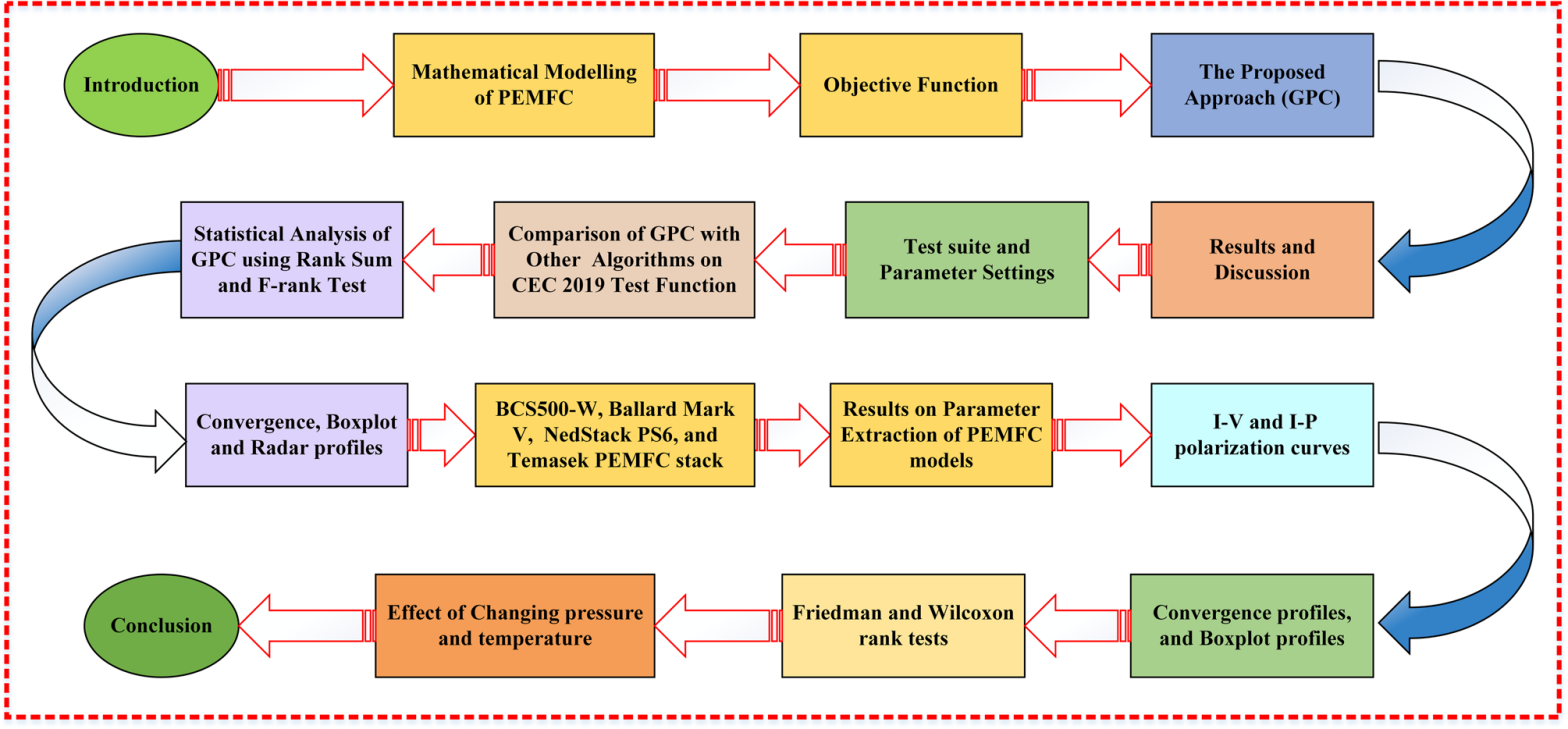
Graphical outline of the article.

This paper’s main contributions can be summarized as follows.

- To precisely estimate the parameters of the PEMFC, a hybridized algorithm known as the Grey Particle Cuckoo (GPC) algorithm has been presented and validated.
- The GPC algorithm has been tested with CEC 2019 challenges and compared to BWOA^65^, CDO^58^, COA^59^, FPA^66^, HHO^60^, YDSE^55^, ZOA^51^, ARNMRA^67^, FROBLGJO^68^, and jDE100^69^. Also, non-parametric test (Friedman and Wilcoxon signed rank test) analysis, as well as the box plot, have been conducted to verify the precision as well as reliability of the GPC algorithm in comparison to existing MH algorithms.
- Four different commercial FC stacks (NedStack PS6, Ballard Mark V, Temasek, as well as the BCS 500 W PEMFC model) have been evaluated to assess the accuracy as well as reliability of the GPC algorithm.
- Comparing the PEMFC results obtained from the proposed GPC algorithm with other MH algorithms (ZOA^51^, SCHO^52^, PSA^53^, SABO^54^, YDSE^55^, EDO^56^, RIME^57^, CDO^58^, COA^59^, HHO^60^, and GWO^61^), it was evident that the GPC algorithm performed significantly.
- In addition, the GPC algorithm is applied for an optimal analysis of the PEMFC stacks with changing pressure ($P_{H2}$ / $P_{O2}$) and temperature levels.
- Statistical studies such as SSE, IAE, MBE, MAE, MSE and RMSE, as well as nonparametric test (Friedman and Wilcoxon signed rank test) have been performed to demonstrate the superiority of the GPC algorithm compared to the other eleven MH optimization algorithms.

The structure of the paper is as follows. Section II outlines the mathematical concept of a PEMFC and the optimization challenge of identifying the unknown parameters of a PEMFC. The details of the proposed approach are presented in Section III. Section IV presents the experimental results as well as a related discussion. The conclusion and future scope of the presented work are given in Section V. The graphical outline of the article is given in Fig. 1.

## Mathematical modeling of PEMFC

**Fig. 2:**
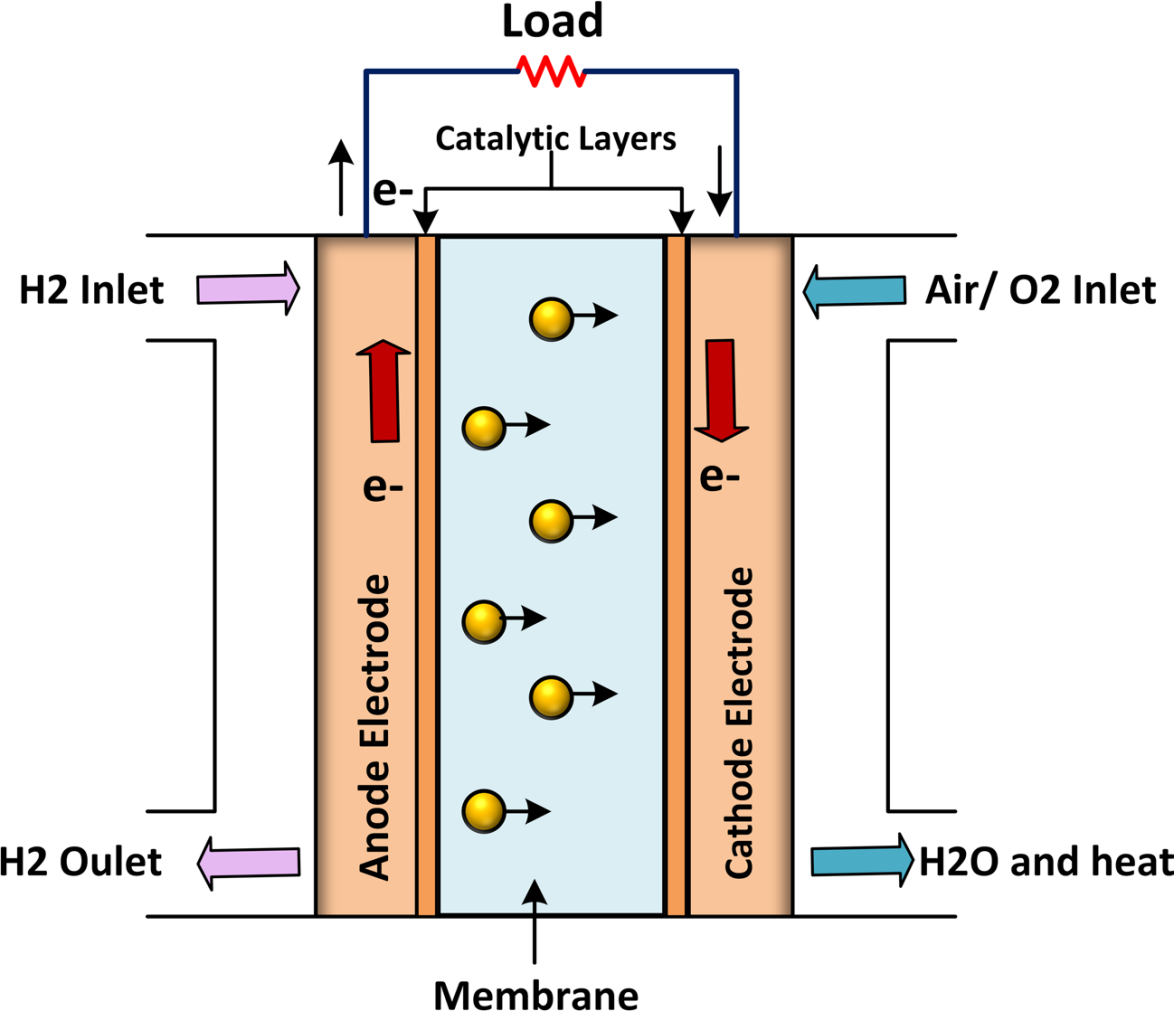
PEMFC model.

A PEMFC is made up of two electrodes, an anode as well as a cathode, with a thin solid membrane that conducts protons placed between them^70,71^, as illustrated in Fig. 2. In addition, Fig. 2 illustrates the reactions that take place at two electrodes. In the catalyst layer of the cathode, the oxygen reacts with the electrons and protons, resulting in the production of water and electricity. The overall reaction is given below:

1 $$\begin{aligned} H_{2}+\frac{1}{2}O_{_{2}}\overset{pt}{\rightarrow }H_{2}O+electricity+heat \end{aligned}$$

**Fig. 3:**
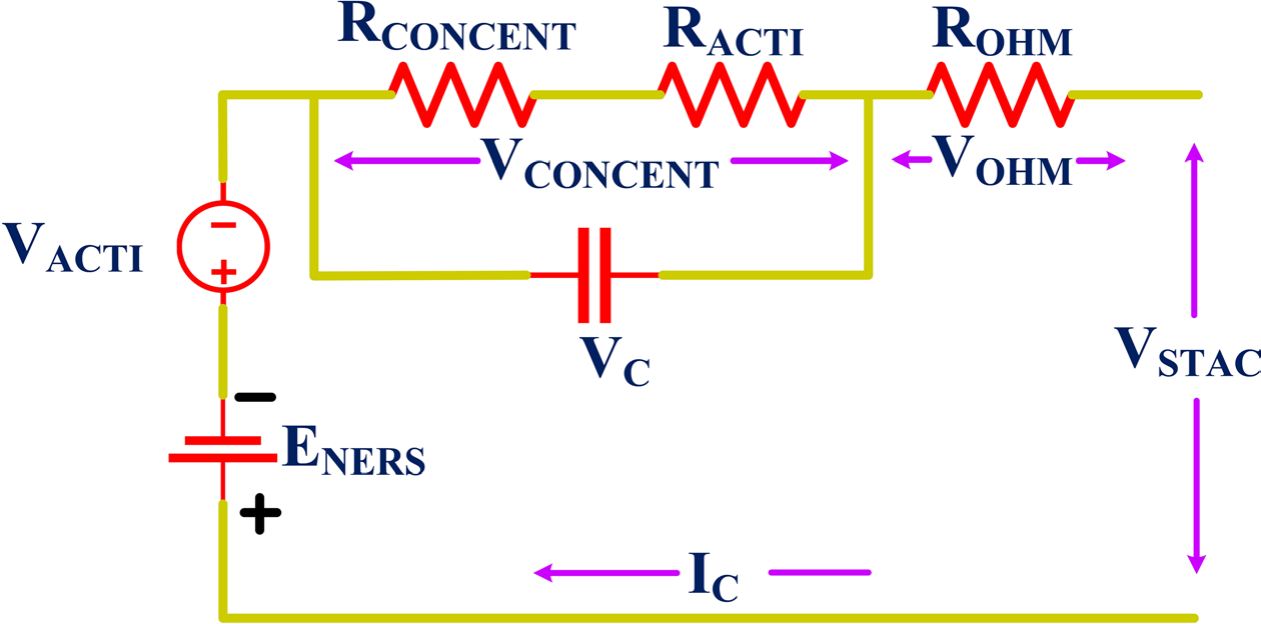
PEMFC equivalent circuit.

The electrochemical model is utilized to mathematically show the behaviour of the electrolyzer. Also, the PEMFC equivalent circuit diagram is presented in Fig. 3. The mathematical representation for the output voltage ($V_{STAC}$) of the stack, as shown in Equation 2, consists of many cells connected in series ($N_{num}$)^72,73^.

2 $$\begin{aligned} {{V}_{STAC}}={{N}_{num}}\left[ {{E}_{NERS}}-{{V}_{ACTI}}-{{V}_{OHM}}-{{V}_{CONCENT}} \right] \end{aligned}$$

Where, reversible open circuit voltage (${E}_{NERS}$), activation voltage loss (${V}_{ACTI}$) due to the activation of both the anode and cathode, concentration over-potential (${V}_{CONCENT}$), and ohmic voltage loss (${V}_{OHM}$). The ${E}_{NERS}$ is determined using the Nernst equation, as shown in Eq. (3)^29^.

3 $$\begin{aligned} {{E}_{ner}}=1.229-8.5*{{10}^{-04}}\left[ {T_{ot}}-298.15 \right] + 4.3085*{{10}^{-05}}* {T_{ot}}\left[ \ln \left( P_{H2}+\sqrt{P_{O2}} \right) \right] \end{aligned}$$

where the cell operating temperature (${T_{ot}}$), partial pressure (atm) of hydrogen and oxygen ($P_{H2}$ as well as $P_{O2}$), can be calculated using Eq. (4), as well as (5)^74,75^.

4 $$\begin{aligned} & P_{H2}=0.5\times {RH}_a\times P_{{H_2}O}\left[ \left( exp\left( \frac{1.635\left( \frac{I_{oc}}{A}\right) }{{T_{ot}}^{1.334}}\right) \times \frac{{RH}_a\times P_{{H_2}O}}{P^a}\right) ^{-1} -1\right] \end{aligned}$$

5 $$\begin{aligned} & P_{O2}={RH}_c\times P_{{H_2}O}\left[ \left( exp\left( \frac{4.192\left( \frac{I_{oc}}{A}\right) }{{T_{ot}}^{1.334}}\right) \times \frac{{RH}_a\times P_{{H_2}O}}{P^c}\right) ^{-1}-1\right] -1 \end{aligned}$$

Where, the inlet pressure of the cathode as well as the anode ($P^c$ and $P^a$), saturation pressure of water vapor ($P_{{H_2}O}$), operating current ($I_{oc}$), and PEM area (*A*).

The $V_{ACTI}$, which is the $2^{nd}$ term on the right side of Eq. (2), can be computed utilizing the Eq. (6)^71^.

6 $$\begin{aligned} V_{ACTI}=-\left[ \xi _a+\xi _b*T_{ot}+\xi _c*T_{ot}*l\ n\left( C_{O2}\right) + \xi _d*T_{ot}*l\ n\left( I_c\right) \right] \end{aligned}$$

Where, semi-empirical coefficients ($\xi _a$, $\xi _b$, $\xi _c$, and $\xi _d$), the concentration of oxygen ($C_{O2}$) and is defined as Eq. (7)^40,76^.

7 $$\begin{aligned} C_{O2}= \frac{P_{O2}}{5.08\times {10}^6\times exp(\frac{-498}{T_{ot}})} \end{aligned}$$

The $V_{OHM}$, which is the term $3^{rd}$ on the right side of Eq. (2), can be mathematically represented in Eq. (8)^77,78^.

8 $$\begin{aligned} V_{OHM}=I_{oc}\left[ R_{memb}+R_{con}\right] \end{aligned}$$

Where, the resistance of the connections ($R_{con}$), as well as the membrane resistance ($R_{memb}$), that can be obtained by utilizing Eq. (9).

9 $$\begin{aligned} R_{memb}= \rho _{memb}\left( \frac{l_{thic}}{A} \right) \end{aligned}$$

where, the thickness of the membrane ($l_{thic}$), as well as the specific resistivity of the membrane ($\rho _{memb}$), can be calculated using Eq. (10).

10 $$\begin{aligned} \rho _{memb}= \frac{\left( 181.6000\left[ 1+ 0.0620\left( \frac{T_{ot}}{303} \right) ^{2}*J^{2.500}+0.0300* J ^{2} \right] \right) }{\left( \left[ \lambda - 3.0000*J-0.6340 ) \right] exp\left( 4.1800\left( \frac{T_{ot}-303}{T_{ot}} \right) \right) \right) } \end{aligned}$$

Where, actual current density (*J*), and parameter $\lambda$ are adjustable and are associated with the water content of the membrane.

The drop in concentration voltage ($V_{CONCENT}$) is caused by changes in the concentration of reactants on the electrode surface and can be mathematically expressed in Eq. (11):

11 $$\begin{aligned} V_{CONCENT}= -\beta *ln\left[ 1-\left( \frac{J}{J_{max}} \right) \right] \end{aligned}$$

Where, the symbol $\beta$ serves to represent the semi-empirical coefficient.

The main objective of our investigation is to determine the most optimal values for the parameters ($\xi _a$, $\xi _b$, $\xi _c$, $\xi _d$, $R_{con}$, $\lambda$ and $\beta$) through the application of the GPC algorithm. This helps ensure that the output voltage of the model aligns with the experimental data.

### Objective Function

The Eqs. (2–11), present a set of equations where the operation parameters $T_{ot}$, $RH_c$, $P^c$, $RH_a$, $P_{H2}$, $P^a$, as well as $P_{O2}$ are measurable and their values depend on the specific operating conditions. Additionally, the physical parameters ($\xi _a$, $\xi _b$, $\xi _c$, $\xi _d$, $R_{con}$, $\lambda$, and $\beta$) are unknown. Due to the significant impact of the unknown parameter on the model outcomes, it is essential to extract them with the greatest accuracy to be precisely matched with the actual voltage-current (V-I) characteristic of the PEMFC.

Before determining the unknown parameter ($\xi _a$, $\xi _b$, $\xi _c$, $\xi _d$, $R_{con}$, $\lambda$, and $\beta$), it is imperative to determine an objective function. In order to compare with previous literature, the objective of optimization in this study is to determine a set of parameter values that will reduce the SSE between the experimental voltage ($V_{exper}$) as well as the model-estimated voltage ($V_{estimat}$) as determined by Eq. (12).

12 $$\begin{aligned} Minimize (OF): F_{obj} (SSE) = \sum _{k=1}^{N_{volt}}[V_{exper} - V_{estimat}]^{2} \end{aligned}$$

Where, the number of voltage data samples ($N_{volt}$), and the proposed constraints are presented as.

13 $$\begin{aligned} S.t\left\{ \begin{matrix} {{\xi }_{i,\min }}\le {{\xi }_{i}}\le {{\xi }_{i,\max }}{{\forall }_{i}}\in \left\{ a,b,c,d \right\} \\ {{\lambda }_{\min }}\le \lambda \le {{\lambda }_{\max }} \\ {{\beta }_{\min }}\le \beta \le {{\beta }_{\max }} \\ {{R}_{con,\min }}\le {{R}_{con}}\le {{R}_{con,\max }} \\ \end{matrix} \right. \end{aligned}$$

In the next section, we present the basics of nature-inspired algorithms and proposed methodology used to optimize the objective function discussed above.

## Basics of nature-inspired algorithms

This section presents the fundamental principles underlying the algorithms utilized to develop a novel GPC optimization algorithm. This is an outline of the recently employed algorithms, including GWO, PSO, and CS optimization algorithms:

### Grey Wolf Optimizer

In 2014, Mirjalili et al. proposed the GWO algorithm, inspired by the social behaviour as well as hunting strategies of wild grey wolves, scientifically known as Canis lupus^61^. These wolves reveal social behaviour and maintain a rigid social hierarchy, categorised into four distinct ranks: alpha ($\alpha$), beta ($\beta$), delta ($\delta$), as well as omega ($\omega$). The mathematical framework of GWO depends on the social structure and hunting techniques of grey wolves. The fundamental aspects of hunting involve tracking, encircling, and subsequently attacking the prey^79^.

#### Social hierarchy

In the development of the GWO, the social hierarchy of wolves is mathematically expressed by identifying the optimal solution as the $\alpha$. Therefore, the $2^{nd}$ and $3^{rd}$ most efficient solutions are designated as $\beta$ as well as $\delta$ accordingly. The remaining candidate solutions have been assumed to be $\omega$. The optimization process of the GWO algorithm is governed by $\alpha$, $\beta$, and $\delta$. The $\omega$ wolves follow the group of 3 wolves ($\alpha$, $\beta$, and $\delta$).

#### Encircling prey

Grey wolves encircle their prey while hunting, as mentioned earlier. In order to represent encirclement behaviour numerically, the following equations are given.

14 $$\begin{aligned} & \overset{\rightarrow }{\mathop {R}}\,=\left| \overset{\rightarrow }{\mathop {E}}.\overset{\rightarrow }{\mathop {{{T}_{p}}}}\,(t)-\overset{\rightarrow }{\mathop {T}}\,(t) \right| \end{aligned}$$

15 $$\begin{aligned} & \overset{\rightarrow }{\mathop {T}}\,(t+1)={{\overset{\rightarrow }{\mathop {T}}\,}_{p}}(t)-\overset{\rightarrow }{\mathop {Q}}.\overset{\rightarrow }{\mathop {R}}\ \end{aligned}$$

where variables $\overset{\rightarrow }{\mathop {T}}\,(t+1)$, *t*, and $\overset{\rightarrow }{\mathop {T}}\,_{p}(t)$ denote the position of the $i^{th}$ grey wolf, the current iteration, and the location of the prey, respectively. The vectors $\overset{\rightarrow }{\mathop {Q}}$ and $\overset{\rightarrow }{\mathop {E}}$ acting as control parameters are computed using Eqs. (16) and (17).

16 $$\begin{aligned} & \overset{\rightarrow }{\mathop {Q}}\,=2\overset{\rightarrow }{\mathop {b}}.\overset{\rightarrow }{\mathop {{{r}_{1}}}}\,-\overset{\rightarrow }{\mathop {b}} \end{aligned}$$

17 $$\begin{aligned} & \overset{\rightarrow }{\mathop {E}}\,=2.\overset{\rightarrow }{\mathop {{{r}_{2}}}} \end{aligned}$$

where components $\overset{\rightarrow }{\mathop {b}}$ are linearly decreased from 2 to 0 throughout the iterations, while $\overset{\rightarrow }{\mathop {{{r}_{1}}}}$ and $\overset{\rightarrow }{\mathop {{{r}_{2}}}}$ are random vectors within the interval [0, 1].

#### Hunting

Grey wolves identify the location of prey and encircle it, with direction from the $\alpha$. The $\beta$ and $\delta$ may also engage in the hunt. The optimum location (prey) remains unknown. To model wolf hunting behaviour, it is assumed that the $\alpha$, $\beta$, and $\delta$ possess superior knowledge regarding potential prey locations. Retain the three best solutions and adjust the positions of other search agents based on the positions of the top-performing agents.

18 $$\begin{aligned} & \overset{\rightarrow }{\mathop {{{R}_{\alpha }}}}\,=\left| \overset{\rightarrow }{\mathop {{{E}_{1}}.}}\,\overset{\rightarrow }{\mathop {{{T}_{\alpha }}}}\,-\overset{\rightarrow }{\mathop {T}}\, \right| ;\overset{\rightarrow }{\mathop {{{R}_{\beta }}}}\,=\left| \overset{\rightarrow }{\mathop {{{E}_{2}}.}}\,\overset{\rightarrow }{\mathop {{{T}_{\beta }}}}\,-\overset{\rightarrow }{\mathop {T}}\, \right| ;\overset{\rightarrow }{\mathop {{{R}_{\delta }}}}\,=\left| \overset{\rightarrow }{\mathop {{{E}_{3}}.}}\,\overset{\rightarrow }{\mathop {{{T}_{\delta }}}}\,-\overset{\rightarrow }{\mathop {T}}\, \right| \end{aligned}$$

19 $$\begin{aligned} & \overset{\rightarrow }{\mathop {{{T}_{1}}}}\,=\overset{\rightarrow }{\mathop {{{T}_{\alpha }}}}\,-\overset{\rightarrow }{\mathop {{{Q}_{1}}}}.(\overset{\rightarrow }{\mathop {{{R}_{\alpha }}}}\,);\overset{\rightarrow }{\mathop {{{T}_{2}}}}\,=\overset{\rightarrow }{\mathop {{{T}_{\beta }}}}\,-\overset{\rightarrow }{\mathop {{{Q}_{2}}}}.(\overset{\rightarrow }{\mathop {{{R}_{\beta }}}}\,);\overset{\rightarrow }{\mathop {{{T}_{3}}}}\,=\overset{\rightarrow }{\mathop {{{T}_{\delta }}}}\,-\overset{\rightarrow }{\mathop {{{Q}_{3}}}}.(\overset{\rightarrow }{\mathop {{{R}_{\delta }}}}\,) \end{aligned}$$

20 $$\begin{aligned} & \overset{\rightarrow }{\mathop {T}}\,(t+1)=\frac{\overset{\rightarrow }{\mathop {{{T}_{1}}}}\,+\overset{\rightarrow }{\mathop {{{T}_{2}}}}\,+\overset{\rightarrow }{\mathop {{{T}_{3}}}}\,}{3} \end{aligned}$$

#### Attacking the prey

Grey wolves finalise the hunt by focussing on the prey once it becomes stationary. To build a mathematical model of the hunter advancing towards its prey, decrease the value of $\overset{\rightarrow }{\mathop {b}}$. The range of $\overset{\rightarrow }{\mathop {{{Q}}}}$ decreases by the effect of $\overset{\rightarrow }{\mathop {b}}$. $\overset{\rightarrow }{\mathop {{{Q}}}}$ is a randomly chosen value within the interval of -2b to 2b, where $\overset{\rightarrow }{\mathop {b}}$ is progressively decreased from 2 to 0 during the repetitions. When random values of $\overset{\rightarrow }{\mathop {{{Q}}}}$ range from [-1,1], the subsequent location of a search agent may lie anywhere between its current location and the location of the prey. The coefficient$\overset{\rightarrow }{\mathop {T}}$ additionally regulates the exploratory phase of the algorithm. This component allocates arbitrary weights to prey to avoid stagnation at local optima, enabling the algorithm to incorporate randomization during the optimization process. In doing so, grey wolves engage in a hunting process characterized by repetitive behaviors of encircling and pursuing, as mentioned above.

### Particle Swarm Optimization

The PSO is a stochastic optimization method based on population dynamics. It was $I^{st}$ proposed by Kennedy and Eberhart in 1995, drawing inspiration from the social behaviours exhibited in bird flocking and fish schooling^80^.

#### Mathematical formulation

In PSO, a particle is characterised by its location as well as velocity within a *d*-dimensional search space. Let $T_i(t)= (T_{i1 },T_{i2}..........T_{id})$ be the location of particle *i* at iteration (*t*), and velocity $U_i(t)= (u_{i1 },u_{i2}..........u_{id})$. The individual optimal location of the particle is $k_i(t)= (k_{i1 },k_{i2}..........k_{id})$, while the global best location identified by the entire swarm is $k_g(t)= (k_{g1 },k_{g2}..........k_{gd})$. The Eqs. (21), and (22) determine the updates for velocity as well as location:

21 $$\begin{aligned} & u_{i,t+1}^{d}=u_{i,t}^{d}+L1*rand*\left( k_{i,t}^{d}-t_{i,t}^{d} \right) +L2*rand*\left( k_{g,t}^{d}-t_{i,t}^{d} \right) \end{aligned}$$

22 $$\begin{aligned} & t_{i,t+1}^{d}=t_{i,t}^{d}+u_{i,t+1}^{d} \end{aligned}$$

The formulation of a new velocity update equation follows from the addition of inertia weight (*S*) to the velocity update formula.

23 $$\begin{aligned} & u_{i,t+1}^{d}=S*u_{i,t}^{d}+L1*rand*\left( k_{i,t}^{d}-t_{i,t}^{d} \right) +L2*rand*\left( k_{g,t}^{d}-t_{i,t}^{d} \right) \end{aligned}$$

24 $$\begin{aligned} & S(t)=S_{max}-\frac{t}{T_{max}}\left( S_{max}-S_{min} \right) \end{aligned}$$

where, *t* and $T_{max}$ are the current iteration as well as maximum iteration. The $S_{max}$ and $S_{min}$ indicate the maximum as well as lower limits of the range of inertia weight (*S*(*t*)) parameter.

### Cuckoo search algorithm

The CS algorithm has been motivated by the obligatory brood behavior of cuckoos as well as relies on three fundamental principles such as.

- Each cuckoo lays one egg at a time, depositing it in a randomly selected nest.
- The nests with the best quality eggs (i.e., best fitness solutions) are carried over to the next generation.
- There are a fixed number of host nests, and there is a probability that the worst nests will be replaced by new ones, representing the discovery of alien eggs by host birds.

The CS algorithm primarily focuses on exploration as well as exploitation of cuckoo species, as outlined by these three principles. The process is divided into two main stages: a local search stage that addresses exploitation and a global search stage that deals withexploration. Another parameter functions as the governing element of the CS algorithm. The parameter selected randomly from a uniform distribution is referred to as the switch probability, denoted as *p*. The subsequent subsections provide an expanded discussion of each of the previously covered stages.

#### Global search phase

The cuckoo search has been executed in accordance with the three rules. A Lévy flight is executed to provide a new solution Y for the ith cuckoo. This process is referred to as a global random walk and is outlined in Eq. (25)

25 $$\begin{aligned} T_{i}^{p+1}=T_i^p+\alpha \otimes {L(\lambda )}(T_{best}-T_i^p) \end{aligned}$$

where as $T_i^p$ denotes the previous solution, while $T_{i}^{p+1}$ is current soloution. This step follows the newly generated solutions using Lévy flights ($L(\lambda )$). The main reason for using such this mechanism is the longer tail and better flight trajectory of the Lévy flight mechanism, which helps to provide better search capabilities to the algorithm. Apart from that, the Lévy flight mechanism is given in Eq. (26).

26 $$\begin{aligned} \ L(\lambda )\sim \frac{\lambda \Gamma (\lambda )\sin (\pi \lambda /2)}{\pi }\frac{1}{s^{1+\lambda }} \hspace{10pt}(s\gg s_0\gg 0) \end{aligned}$$

where $\hspace{5pt} s=\frac{U}{|V|^1/\lambda }\hspace{10pt} U\sim N(0,\sigma ^2), \hspace{10pt}V\sim N(0,1)$ and $\sigma ^2=\bigg \{\frac{\Gamma (1+\lambda )}{\lambda \Gamma [(1+\lambda )/2]}. \frac{\sin (\pi \lambda /2)}{2^{(\lambda -1)/2}}$. Also, $\Gamma (\lambda )$ is a gamma function and the value of $\lambda$ is equal to 1.5. During this exploration phase, the parameter *N* is sampled from a standard Gaussian distribution with a mean of 0 and a variance of $\sigma ^2$. This process is designed to explore the solution space effectively. To generate a new solution, the current best solution ($T_{best}$) is utilised in conjunction with the sample parameter.

#### Local search phase

The $2^{nd}$ phase of CS algorithm is the local random-walk mechanism, which aligns with the exploitation process. This phase involves the generation of a new solution ($T_i^{p+1}$) through a local search using two randomly selected solutions from the search pool.The local random walk is presented in Eq. (27).

27 $$\begin{aligned} T_{i}^{p+1}=T_i^p+\alpha \otimes (\epsilon )\otimes (T_j^p-T_k^p) \end{aligned}$$

where $T_j^p$ and $T_k^p$ correspond to two random solutions, $\epsilon \in [0, 1]$ is a uniformly distributed random number.

## The proposed approach

This section deals with the proposal of the GPC algorithm, starting with the motivation behind the proposal, the details of the proposal and finally the computational complexity of the proposed approach.

### Motivation behind the proposal

In optimization, the trade-off between searching for new, potentially better solutions (exploration) and refining known good solutions (exploitation) is a critical determinant of algorithmic success. Exploration enables coverage of diverse and unexplored regions in the search space, increasing the likelihood of escaping local optima, while exploitation ensures refinement of promising solutions for accelerated convergence^81^. Striking an effective balance between these two processes is essential, as overemphasis on either may lead to an inefficient search or premature convergence. Addressing this, the proposed GPC algorithm adopts a modular, phase-wise hybridization strategy to enforce a temporal and spatial balance between exploration and exploitation.

The algorithm utilizes CS-based L’evy flight updates in the early stages to ensure a wide exploratory radius, leveraging the heavy-tailed distribution to traverse far-reaching regions of the search space. As the algorithm progresses, the leadership-driven model of GWO is introduced to promote guided, yet diverse, exploitation through hierarchical decision-making. To further intensify convergence in later iterations, PSO-based velocity updates are incorporated with adaptively tuned inertia weights. This phase-wise hybridization is not a simple combination, but a role-specific assignment, where each component algorithm governs a particular aspect of the search. Unlike traditional hybrids that apply all components uniformly or in a static blend, GPC dynamically partitions the population and allocates algorithms temporally, allowing each strategy to dominate in its optimal phase. This role-based hybridization not only enhances search efficiency, but also reduces algorithmic bias. The adaptability of the framework ensures resilience in diverse problem landscapes, in accordance with the No Free Lunch Theorem^50^. Thus, the novelty of GPC lies in the structured orchestration and transition of exploration–exploitation roles, rather than merely the selection of constituent algorithms.

### The Grey Particle Cuckoo Algorithm

Although individual metaheuristic algorithms such as PSO^80^, GWO^61^ and CS^82^ have demonstrated merit in solving complex optimization problems, each suffers from specific limitations. PSO is prone to premature convergence due to loss of diversity and stagnation in local optima. GWO lacks exploratory capabilities in the early stages and tends to focus narrowly on leader-driven search. CS, despite its exploratory strength through Lévy flights, exhibits instability in convergence and often fails to fine-tune solutions effectively. An inherent limitation of these basic algorithms lies in their suboptimal exploration capability, given their tendency to follow smaller step sizes. This behavior leads to the clustering of new solutions, impeding the exploration process. Consequently, the algorithm tends to confine its search to specific regions within the search space, resulting in an inefficient approach to exploration and an overall sub-par operational performance. Although there are problems with basic algorithms, the organizational structure of CS has been found to be very efficient and can be used as a benchmark to propose new algorithms^81^.

To counter these deficiencies, GPC proposes a layered hybridization model that restructures both global and local search dynamics. The global search capability is enhanced by embedding new movement equations that encourage distant but purposeful solution updates. Gleichzeitig, the local exploitation phase is improved through mechanisms that capitalize on the proximity of current best solutions, continuously benchmarking new candidates against elite ones, and accelerating convergence toward global optima. The GPC architecture is designed to preserve the useful traits of its constituents (GWO, PSO, and CS) while mitigating their drawbacks. Retain the organizational efficiency of CS, refine the hierarchical strategy of GWO for better guidance, and incorporate the adaptive velocity control of PSO for convergence tightening. These elements are integrated without disrupting the structural integrity of the parent algorithms, making GPC an inherently robust and versatile optimization framework. We now formally define the algorithmic steps, population management, and GPC control parameters.

The algorithm begins by setting *N* within a constrained search space, as defined by (29)

28 $$\begin{aligned} y_{i,0}^j=y_{min}^j+ a(y_{max}^j-y_{min}^j)\hspace{10pt} j=1,2,...,D \end{aligned}$$

for the $i^{th}$ member of the search space, *j* is the *D* dimensional problem, $a \in [0, 1]$ $y_{min}$ and $y_{max}$ are the lower bound and upper bounds of the $D^{th}$ problem. After initialization, we divide the population into two iterative halves.

#### Stage I : $p = 1: \frac{p_{maximum}}{2}$

In the first step, we follow the basic search equations of CS algorithm. This step follows the newly generated solutions using Lévy flights. The main reason for utilizing this mechanism is the longer tail and better flight trajectory of the Lévy flight mechanism, which helps to provide better search capabilities to the algorithm. The general equation for this stage is given by

29 $$\begin{aligned} z_{i}^{p+1}=z_i^p+\alpha \otimes {L(\lambda )}(z_{best}-z_i^p) \end{aligned}$$

where

Apart from that, the Lévy flight mechanism is given by

30 $$\begin{aligned} \ L(\lambda )\sim \frac{\lambda \Gamma (\lambda )\sin (\pi \lambda /2)}{\pi }\frac{1}{s^{1+\lambda }} \hspace{10pt}(s\gg s_0\gg 0) \end{aligned}$$

The local search phase is the local random-walk mechanism, which aligns with the exploitation process. This phase involves the generation of a new solution ($z_i^{p+1}$) through a local search using two randomly selected solutions from the search pool. The overarching equation for the local search phase is provided as follows:

31 $$\begin{aligned} z_{i}^{p+1}=x_i^p+\alpha \otimes (\epsilon )\otimes (z_j^p-x_k^p) \end{aligned}$$

where $z_j^p$ and $z_k^p$ correspond to two random solutions, $\epsilon \in [0, 1]$ is a uniformly distributed random number.

The local search phase is activated only for a certain fraction of iterations and depends on the probability switch parameter (*sp*). In the current scenario, this parameter is adjusted using an exponential decreasing inertia weight operator. This phase helps to decide the extent of exploration and exploitation. The general equation is thus given by

32 $$\begin{aligned} sp(p)=\eta _{min}+(\eta _{max}-\eta _{min})exp\left[ -\frac{p}{(\frac{p_{max}}{10})}\right] \end{aligned}$$

where $\eta _{max}$ as well as $\eta _{min}$ are maximum and minimum random values [0,1].

#### Stage II : $p = \frac{p_{maximum}}{2}+ 1: p_{maximum}$

In this phase, the main concern is to have lower exploration and more exploitation. During this phase, the global search phase is divided into two parts, and here also the first half of the population follows a similar approach as used in the generalized algorithm. This is done to make the algorithm perform intensive exploration along with some exploitation operation. For the second half of the population, GWO based equations are used. These equations are meant for the initialization of new solutions within the search space, are generally generated using three new solutions, and are formulated as

33 $$\begin{aligned} & f_{Cauchy(0,h)}(\delta )=\frac{1}{\pi } \frac{h}{({h^2}+{\delta ^2})} \end{aligned}$$

34 $$\begin{aligned} & y=\frac{1}{2}+\frac{1}{\pi }arctan(\frac{\delta }{h}) \end{aligned}$$

35 $$\begin{aligned} & \delta =tan(\pi (y-\frac{1}{2})) \end{aligned}$$

36 $$\begin{aligned} & z_i^{p+1}=z_i^p+\alpha \times {D(\delta )}(z_{best}-z_i^p) \end{aligned}$$

37 $$\begin{aligned} & \begin{aligned} z_1=z_i-J_1(H_1.z_{new}-z_i^p);\\ z_2=z_i-J_2(H_2.z_{new}-z_i^p);\\ z_3=z_i-J_3(H_3.z_{new}-z_i^p) \end{aligned} \end{aligned}$$

38 $$\begin{aligned} & z_i^{p+1}=\frac{z_1+z_2+z_3}{3} \end{aligned}$$

39 $$\begin{aligned} & N=2b.r_1-b; \hspace{5pt} N=2.r_2 \end{aligned}$$

During this phase, the local search equation is further categorized into two distinct population segments. For the $I^{st}$ half of the population, again CS based local search is followed, but for the second half of the population, a more rigorous approach is used. Here we are using a PSO-based equation as given by

40 $$\begin{aligned} z_{i}^{t+1}=z_i^t + F \times (z_{best}-z_i^t) + I \times (g_{best}^t-z_i^t) \end{aligned}$$

where $g_{best}$ is the personal best solution, $z_{best}$ is the current best solution, $F = c_1.r_1$ is a random number initialized using simulated annealing inertia weight and is given by

41 $$\begin{aligned} c_1= \eta _{min}+(\eta _{max}-\eta _{min})\times a^{(T-1)} \end{aligned}$$

where, the variables *T*, $r_1$ $\eta _{max}$, and $\eta _{min}$ are uniformly distributed in the range [0,1]. Furthermore, the value of *a* is set to 0.95.

$I = c_2.r_2$ is another random number initialized using the sigmoid inertia weight operation as given by

42 $$\begin{aligned} & c_2= \frac{\eta _{min}-\eta _{max}}{1+e^{-u\times (p-h\times gen)}}+\eta _{max} \end{aligned}$$

43 $$\begin{aligned} u=10^{\log (gen)-2} \end{aligned}$$

where, $$\eta _{max}=0.9, \eta _{min}=0.5$$, $$gen=51$$, $$r_2$$ and $$h, k \in [0, 1].$$

#### Population adaptation

To address a reduction in population size, a strategy outlined in^82^ is followed. The proposed approach involves modifying the population size during algorithm iterations, emphasizing greater exploration initially and transitioning towards adaptive exploitation later. The reduction in population size as iterations progress aims to optimize the algorithm by efficiently utilizing search agents. In simpler terms, the algorithm employs a larger population during exploration to thoroughly cover the search space, while exploitation, focused on specific areas, is achieved with a smaller population. This adaptive population adjustment ensures effective exploration in the early stages and targeted exploitation in the later stages, striking a balance between resource utilization and optimization.

In this study, a proportional population reduction phenomenon is employed, inspired by the ideas presented in^82^. This method involves reducing the population size in proportion to the increase in fitness. The rationale behind adopting this approach lies in its suitability for addressing multimodal problems, where solutions need to explore extensive areas. When confronted with a large initial population size, it becomes possible for new solutions to explore the entire search space. As the iterations progress, these solutions may converge toward the global best solution, which lies in a specific direction. Consequently, the size of the population can decrease. This reduction in population size facilitates improved genetic drift, allowing the discovery of new solutions without compromising the retention of the best solution. Toward the conclusion of the process, each member of the population is given an equal opportunity to potentially become the best global solution. In a more general context, the equation governing population reduction is expressed as

44 $$\begin{aligned} Q_{p+1}= {\left\{ \begin{array}{ll} (1-\sigma f_p^{best})n_p, \hspace{10pt} if \hspace{5pt} \sigma f_p^{best}\le \sigma f_{max}^{best}\\ (1-\sigma f_{max}^{best})n_p, \hspace{10pt} if \hspace{5pt} \sigma f_p^{best}> \sigma f_{max}^{best}\\ {min}_{n}, \hspace{48pt} if \hspace{5pt} Q_{p+1} <{min}_{n} \end{array}\right. } \end{aligned}$$

Where, $Q_{p+1}$ represents the population at generation *p*, $\sigma f_p^{best}$ is stated by $(\frac{f_{p-1}^{best}-f_{p-2}^{best}}{|f_{p-2}^{best}|})$ indicates the change in the best fitness, and $\sigma f_{max}^{best}$ presents the threshold value.

#### Selection operation

In the last phase, it is important to carefully select the individuals who are the most appropriate from both the candidates generated in the current iteration and those from previous iterations. The selection technique is determined by comparing the fitness of the new solution, indicated as $f(z_{new})$, with the fitness of the previously known solution, represented as $f(z_i^p)$.If the fitness of the new solution, denoted as ($f(z_{new}$)), is found to be superior to the fitness of the previous known solution, denoted as ($f(z_i^p)$), then the new solution replaces the original solution as the one selected. However, if the previous solution ($z_i$) has a higher level of fitness, it will be retained as the selected solution for the subsequent stages of the process.

45 $$\begin{aligned} z_{new}^{p+1}= {\left\{ \begin{array}{ll} z_{new} \hspace{20pt}if \hspace{5pt} f(z_{new})<f(z_i^p) \\ z_i^p \hspace{35pt} otherwise \end{array}\right. } \end{aligned}$$

**Fig. 4:**
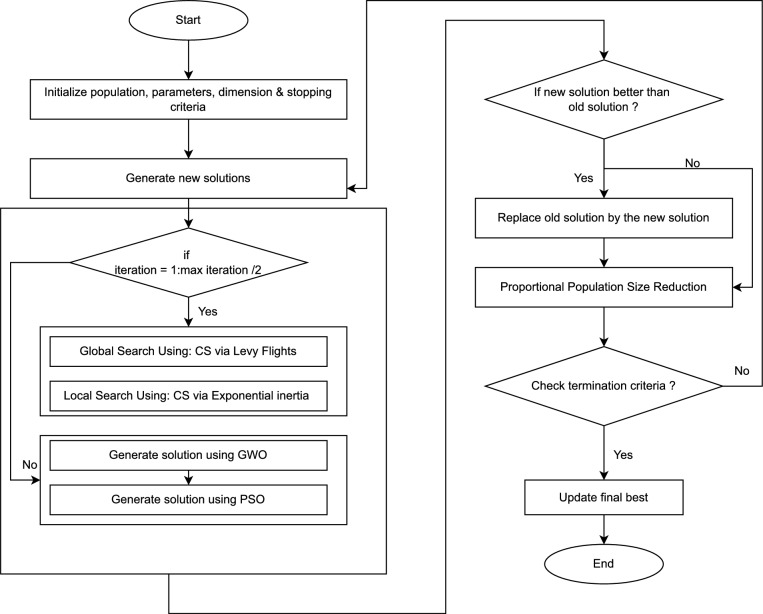
Flowchart of GPC algorithm.

This selection mechanism ensures that the most suitable individuals remain in the population, encouraging improved fitness and ultimately leading to a more optimal and efficient solution for the problems under consideration. The pseudocode of the GPC algorithm is presented in Algorithm 1. The pseudocode of the proposed algorithm is shown in Fig. 4.

**Algorithm 1:**
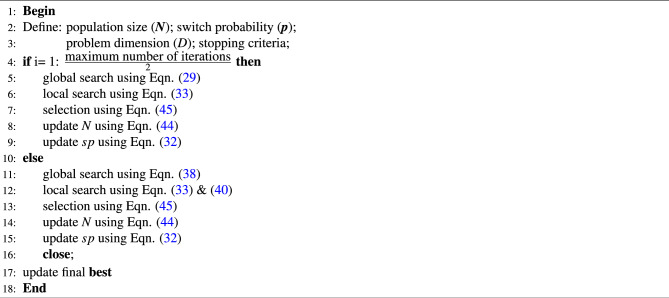
Pseudocode of proposed GPC algorithm.

### Complexity of the proposed GPC algorithm

The computational complexity of the proposed GPC algorithm is governed by the operations performed during initialization and across each iteration of the main loop. Let *N* be the population size, *D* be the dimensionality of the optimization problem, and $t_{\text {max}}$ be the maximum number of iterations.

- **Initialization:** Each of the *N* individuals is initialized in a *D*-dimensional space. This step requires $\mathscr {O}(N \cdot D)$ operations.
- **Stage I (**$t = 1$** to **$t_{\text {max}}/2$): Each individual undergoes a global update based on Lévy flights and, with some probability, a local search using two random solutions. Both operations are $\mathscr {O}(D)$ per individual per iteration, resulting in $\mathscr {O}(N \cdot D)$ per iteration.
- **Stage II (**$t = t_{\text {max}}/2 + 1$** to **$t_{\text {max}}$): The population is divided into two segments. One-half uses GWO-based updates (involving three directional updates and averaging), while the other-half uses a PSO-based update with adaptive inertia weights. Each of these update mechanisms requires $\mathscr {O}(D)$ operations per individual per iteration.

Combining all stages, the total computational complexity of the GPC algorithm is as follows.

46 $$\begin{aligned} \mathscr {O}(N \cdot D + \frac{t_{\text {max}}}{2} \cdot N \cdot D + \frac{t_{\text {max}}}{2} \cdot N \cdot D) = \mathscr {O}(t_{\text {max}} \cdot N \cdot D) \end{aligned}$$

#### Comparison with baseline algorithms

To contextualize the computational efficiency of the proposed GPC algorithm, we compare its complexity with those of the canonical CS, PSO and GWO, as

- CS involves Lévy flight-based position updates and optional random walk-based local search, both operating in *D* dimensions. The total complexity is:
  47 $$\begin{aligned} \mathscr {O}(t_{\text {max}} \cdot N \cdot D) \end{aligned}$$
- PSO updates each particle’s velocity and position based on personal and global best positions. Each update involves *D*-dimensional operations:
  48 $$\begin{aligned} \mathscr {O}(t_{\text {max}} \cdot N \cdot D) \end{aligned}$$
- GWO updates the position of each individual based on three leading solutions ($\alpha$, $\beta$, $\delta$), requiring three vector operations per update:
  49 $$\begin{aligned} \mathscr {O}(t_{\text {max}} \cdot N \cdot D) \end{aligned}$$

Although all four algorithms share the same asymptotic complexity of $\mathscr {O}(t_{\text {max}} \cdot N \cdot D)$, the proposed GPC algorithm incurs higher constant factors due to its dual-phase architecture and hybridization strategy. Specifically, GPC integrates the Lévy flight exploration of CS, the leader-based exploitation of GWO, and the adaptive velocity-driven convergence of PSO. This structured combination leads to increased per-iteration operations, but significantly improves the algorithm’s ability to balance exploration and exploitation. Therefore, despite similar theoretical complexity, GPC achieves superior performance in diverse and complex optimization scenarios leveraging richer update dynamics.

## Results and discussion

This section demonstrates the experimental results to show the effectiveness of the hybrid algorithm (GPC) proposed in this paper. This section is divided into three subsections. In the $I^{st}$ subsection, details of the test suite and parameter settings of all MH algorithms used for comparative analysis are discussed. In the next subsection, the performance of the GPC algorithm is determined by performing experiments on the numerical optimization challenges of CEC 2019. The results of the GPC optimization algorithm are compared with the other MH optimization such as BWOA^65^, CDO^58^, COA^59^, FPA^66^, HHO^60^, YDSE^55^, ZOA^51^, ARNMRA^67^, FROBLGJO^68^, and jDE100^69^. In the third subsection, GPC is applied for parameter extraction of PEMFC models (Temasek, NedStack PS6, Ballard MarkV, and BCS 500W PEMFC model). The effectiveness of the GPC algorithm is compared with various well-recognized MH algorithms, including ZOA^51^, SCHO^52^, PSA^53^, SABO^54^, YDSE^55^, EDO^56^, RIME^57^, CDO^58^, COA^59^, HHO^60^, and GWO^61^.

### Test suite and parameter settings

The implemented Algorithm (GPC) has been executed using the MATLAB R2023b software environment. The computational experiments have been conducted on a laptop equipped with an Intel$\text{\textregistered}$ Core (TM) i5-12500H operating system at a clock speed of 2.50 GHz, x64-based processor, 64-bit operating system, along with 16 GB of RAM with the Windows 11 operating system. In this subsection, the effectiveness of the proposed GPC algorithm in relation to the benchmark challenges of CEC 2019, and real-world challenge (Parameter Extraction of PEMFC models) is evaluated. The present study tests the effectiveness of the proposed GPC algorithm compared to several MH algorithms such as BWOA^65^, CDO^58^, COA^59^, FPA^66^, HHO^60^, YDSE^55^, and ZOA^51^ on benchmark challenges (CEC 2019). For real-world challenges (parameter extraction of PEMFC models), the GPC algorithm is tested and compared with several MH algorithms, including ZOA^51^, SCHO^52^, PSA^53^, SABO^54^, YDSE^55^, EDO^56^, RIME^57^, CDO^58^, COA^59^, HHO^60^, and GWO^61^. The parameter settings for all algorithms were obtained from their respective papers and are displayed in Table 2.

### The CEC 2019 benchmark challenges

The effectiveness and efficiency of the proposed GPC optimization algorithm are tested on CEC 2019 benchmark challenges by statistically measuring the mean values, as well as standard deviation (Std), and comparing them with those obtained with other MH algorithms. Seven MH optimization algorithms such as CDO^58^, COA^59^, BWOA^65^, FPA^66^, HHO^60^, YDSE^55^ and ZOA^51^ were utilized to evaluate and compare the outcomes achieved by the proposed GPC optimization algorithm. To ensure a fair comparison between GPC and other algorithms, all are subjected to population size of 50 as well as the maximum number of iterations = 500 with 51 runs.

**Table 2:** Parametric details of different algorithms.

S.No.	MH Algorithms	Parameters
1	BWOA^65^	procreate rate (pr) = 0.6; mutation rate (mr) = 0.4; cannibalism rate (cr) = 0.44
2	CDO^58^	speed of beta = Rand (1, 270,000) km/s; speed of gamma= Rand (1, 300,000) km/s; radius of radiations propagation = Rand (0, 1); speed of alpha = Rand (1, 16,000) km/s;
3	COA^59^	random real number (*r*) =[0,1]; Integer (*I*) =[1,2]
4	FPA^66^	$\lambda$ = 1.5; $\varepsilon$ = [0,1], $p=0.5$
5	HHO^60^	$r_1, r_2, r_3, and q$ = [0,1]; escaping energy (E) = Linearly decreased from 2 to 0
6	YDSE^55^	Distance between two slits (*d*) = $5 \times 10^{-3}$ m; Wavelength ($\lambda$) = $5 \times 10^{-6}$ m *I* = 0.01 m; Constant value ($\delta$) = 0.38
7	ZOA^51^	constant number (R) = 0.01; probability ($P_S$) = [0,1]; random number(*r*) = [0,1]
8	SCHO^52^	$rand, r_1, r_2, r_3, r_4, r_5, r_6, r_7, r_8, r_9, r_{10}, r_{11}, r_{12}$ = [0,1]; sensitive coefficient *u* = 0.388; sensitive coefficient *m*=0.45; $\varepsilon$ = 0.003
9	PSA^53^	$rand, r_1, r_2, r_3$ = [0,1]
10	SABO^54^	$rand, r_{i,d}$ = [0,1]
11	EDO^56^	$\phi , rand,$= [0,1]; random number (*f*) = [– 1,1]
12	RIME^57^	$r_1$ = [– 1,1];degree of adhesion(*h*)= [0,1]; $r_2$ = [0,1]
13	GWO^61^	$\alpha$ = Linearly decreased from 2 to 0; $rand, r_1, r_2$ = [0,1]
14	GPC	*sp*, *I*, and *F* are self-adaptive

The results presented in Table 3 show that, for challenge $GPC_{1}$, the FROBLGJO algorithm exhibits outstanding results compared to other MH algorithms. The results for challenges $GPC_{2}$, $GPC_{3}$, $GPC_{4}$, $GPC_{5}$, $GPC_{7}$, and $GPC_{9}$, the outcomes obtained from the GPC algorithm demonstrate superior results compared to other MH techniques in terms of mean as well as Std values. The outcomes obtained for challenges $GPC_{6}$, $GPC_{8}$, and $GPC_{10}$, the ZOA algorithm shows outstanding performance compared to the other MH methods. Therefore, based on the results obtained from the experimentation, it can be observed that of the 10 numerical test challenges, the GPC algorithm shows effectiveness in solving 6 challenges. The ZOA algorithm has shown competence in addressing three challenges, but the FROBLGJO method has been successful in addressing only one challenge. The analysis indicates that the GPC algorithm has superior performance in addressing the numerical challenges of CEC 2019. Therefore, it can be concluded that the GPC optimization algorithm is generally the most effective algorithm to address these challenges.

**Statistical testing:**

Furthermore, two statistical tests, the Friedman rank test and the Wilcoxon rank sum test, have been employed in statistical analysis. The statistical results for each test challenge are presented as loss(l), win(w), or tie(t). Here, “win” (w) is used to represent a scenario in which the test algorithm outperforms the GPC algorithm and is denoted by the symbol “+”. On the other hand,, the term “loss” (l) refers to a scenario in which the test algorithm performs worse than the GPC algorithm and is denoted by the symbol “-”. The symbol “=” is used to indicate a tie (t), indicating that both algorithms are statistically similar in relation to each other. The ranking of all algorithms is shown in the third row of Table 3 for every challenge, denoted by *w*/*l*/*t*. Furthermore, the f-rank is determined for every function, and subsequently, the mean of all rankings is given. Every algorithm has received a distinct ranking according to its performance. The mean rank of each algorithm is shown in the $2^{nd}$ last row of Table 3. In addition, an overall f-rank has been computed based on the outcomes of all challenges, displayed in Table 3. The table indicates that the GPC technique has been the most effective, achieving the highest rank ($1^{st}$) among all tested MH algorithms.

**Fig. 5:**
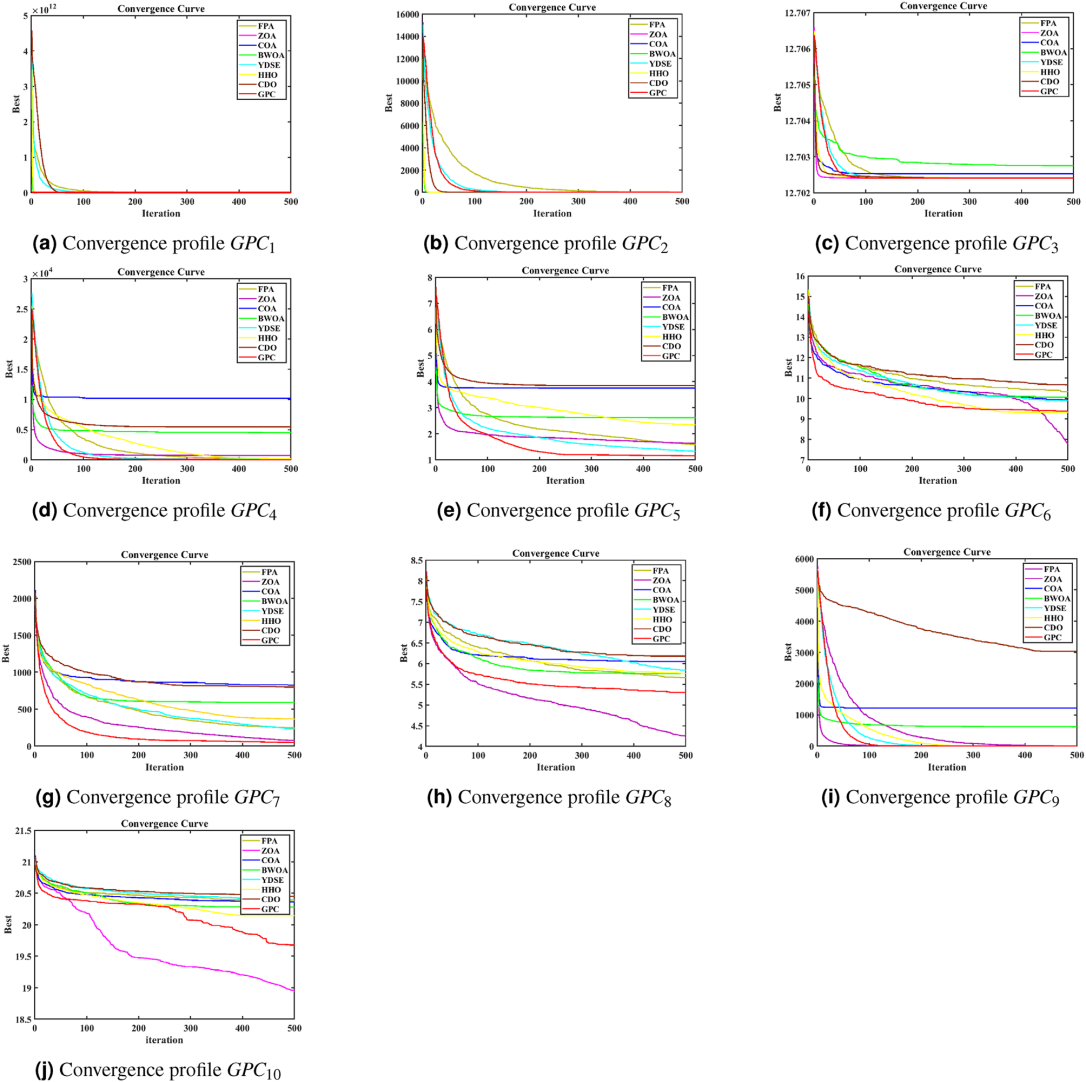
Convergence profiles for the CEC 2019 numerical challenges of FPA, ZOA, COA, BWOA, YDSE, HHO, CDO, and GPC MH algorithms.

**Fig. 6:**
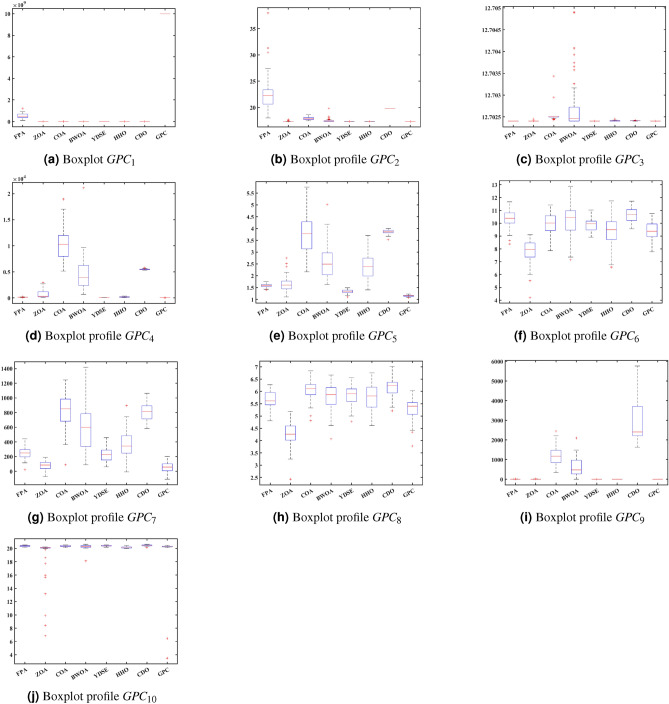
Boxplot profiles for the CEC 2019 numerical challenges for FPA, ZOA, COA, BWOA, YDSE, HHO, CDO, and GPC MH algorithms.

**Fig. 7:**
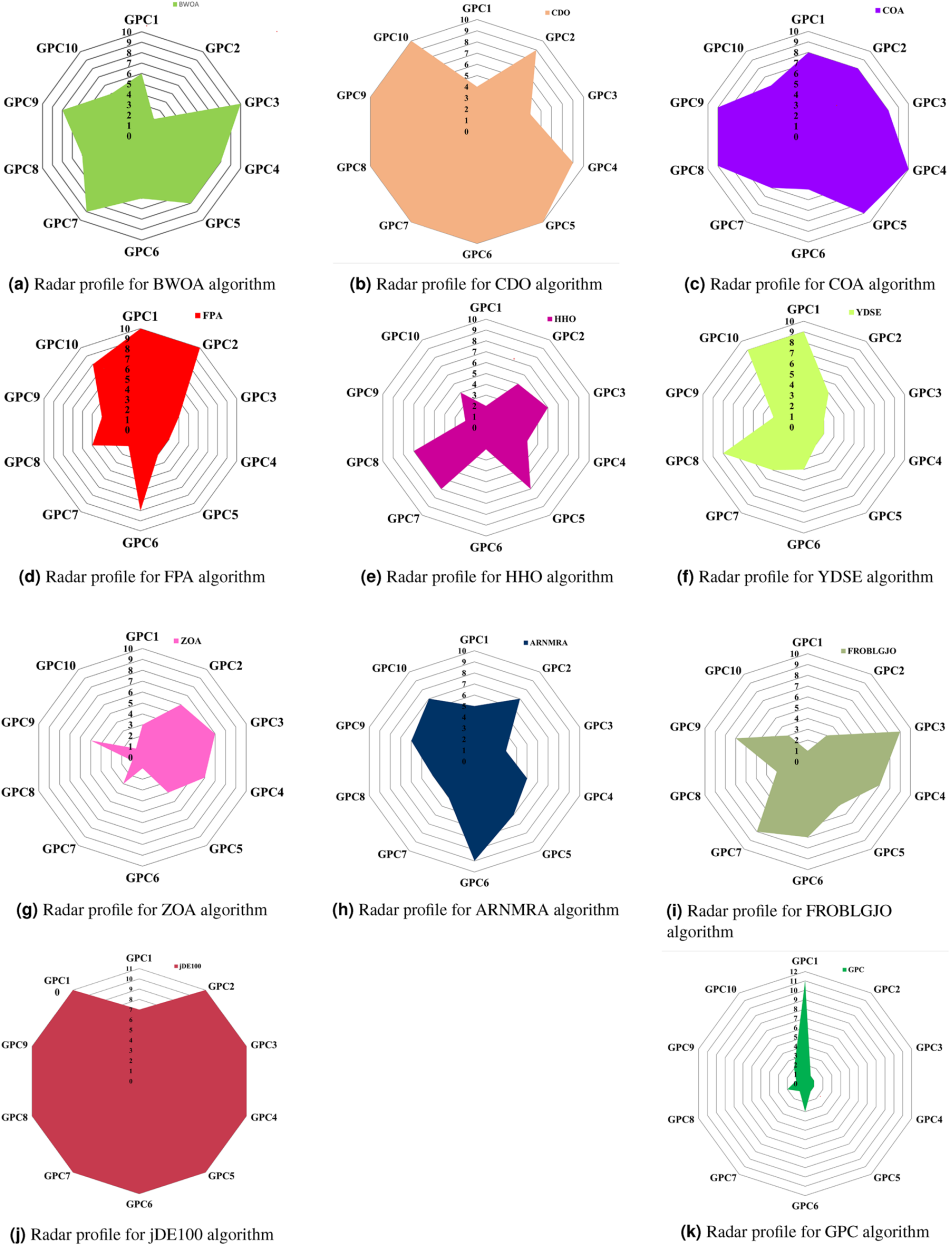
Radar plot for the CEC 2019 numerical challenges of FPA, ZOA, COA, BWOA, YDSE, HHO, CDO, and GPC MH optimization algorithms.

**Convergence profile, Boxplot and Radar profile Analysis:** This subsection displays the convergence, boxplot, and radar profiles of eight MH optimization algorithms such as ZOA, CDO, COA, FPA, BWOA, HHO, YDSE, and GPC. Figures 5, 6, and 7 demonstrate graphic representations. The GPC algorithm exhibits faster convergence for challenges, $GPC_{2}$, $GPC_{3}$, $GPC_{4}$, $GPC_{5}$, $GPC_{7}$, and $GPC_{9}$, and are shown in Fig. 5a–i, as well as 5j, respectively. Figure 6 shows the box plot that represents the fitness values of the COA, YDSE, CDO, HHO, ZOA, FPA, BWOA, and GPC optimization algorithms. The findings indicate that the proposed GPC algorithm is economically efficient regarding fitness values, shown by its significantly low median fitness value. This can be observed from the box plots presented in Fig. 6a–j, respectively. Furthermore, the radar plot in Fig. 7 shows the ranking of the 12 MH optimization algorithms on the CEC2019 test function. The GPC exhibits a smaller darkening area in comparison to the other MH optimization algorithms. This is visible from the radar charts Fig. 7a–k, respectively. The above outcomes demonstrate the performance of the GPC algorithm.

**Table 3:** Statistical outcomes for the 100-digit challenge (CEC 2019) numerical problems.

Challenges		BWOA^65^	CDO^58^	COA^59^	FPA^66^	HHO^60^	YDSE^55^	ZOA^51^	ARNMRA^67^	FROBL-GJO^68^	jDE-100^69^	GPC
$GPC_{1}$	Mean	1.5294E+05	9.5879E+04	1.6522E+05	5.3014E+08	5.0159E+04	3.8512E+06	**7.1435E+04**	1.350E+05	4.520E+04	1.59E+05	1.0000E+10
	Std	2.1936E+05	2.6979E+00	1.26407E+05	2.4666E+08	4.4810E+03	1.8195E+06	**1.4253E+05**	7.839E+04	4.204E+03	1.597E+05	0.0000E+00
	p-rank	+	+	+	+	+	+	+	+	+	+	
	f-rank	6	4	8	10	2	9	3	5	1	7	11
$GPC_{2}$	Mean	1.7532E+01	1.9847E+01	1.7916E+01	**2.2660E+01**	1.7357E+01	1.7355E+01	1.7389E+01	1.784E+01	1.734E+01	2.385E+06	1.7343E+01
	Std	3.8690E–01	0.0000E+00	3.0206E–01	**3.5269E+00**	7.9998E–03	4.9060E–03	9.7529E–01	3.432E–01	8.392E–02	2.719E+04	9.5955E–05
	p-rank	−	−	−	−	−	−	−	−	−	−	
	f-rank	2	9	8	10	5	4	6	7	3	11	1
$GPC_{3}$	Mean	1.2702E+01	**1.2702E+01**	**1.2702E+01**	**1.2702E+01**	**1.2702E+01**	**1.2702E+01**	**1.2702E+01**	**1.270E+01**	**1.270E+01**	1.31E+06	**1.2702E+01**
	Std	6.1579E–04	3.3028E–06	**1.4773E–04**	9.9446E–08	8.4058E–06	6.9331E–10	8.9366E–06	3.245E–08	3.102E–04	8.519E+05	**1.3500E–14**
	p-rank	−	−	−	−	−	−	−	−	−	−	
	f-rank	10	5	8	4	6	2	7	3	9	11	1
$GPC_{4}$	Mean	4.5309E+03	5.4836E+03	**1.0194E+04**	1.3845E+02	1.6806E+02	5.7472E+01	7.3522E+02	3.528E+02	9.776E+02	3.475E+05	4.1084E+01
	Std	3.3190E+03	8.8105E+01	2.361E+02	3.0541E+03	**2.6490E+01**	6.6113E+01	7.9702E+00	8.5208E+02	1.644E+03	1.149E+05	7.7348E+00
	p-rank	−	−	−	−	−	−	−	−	−	−	
	f-rank	8	9	10	3	4	2	6	5	7	11	1
$GPC_{5}$	Mean	2.6193E+00	3.8556E+00	**3.7507E+00**	1.5793E+00	2.3368E+00	1.3300E+00	1.6316E+00	1.939E+00	1.698E+00	1.673e+05	1.1549E+00
	Std	7.0345E–01	8.9450E–02	**7.1965E–01**	8.0326E–02	4.9634E–01	8.1574E–02	3.2080E–01	3.127E–01	4.527E–01	8.426e+04	3.1311E–02
	p-rank	−	−	−	−	−	−	−	−	−	−	
	f-rank	8	10	9	3	7	2	4	6	5	11	1
$GPC_{6}$	Mean	1.0063E+01	1.0667E+01	9.9332E+00	1.0333E+01	9.3245E+00	9.8942E+00	**7.7901E+00**	1.036E+01	1.013E+01	3.841E+04	9.3846E+00
	Std	1.3329E+00	5.6535E–01	8.7596E–01	**7.0873E–01**	1.1888E+00	4.6791E–01	**9.4376E–01**	6.533E–01	1.732E+00	2.063E+03	7.3711E–01
	p-rank	−	−	−	−	+	−	+	−	−	−	
	f-rank	6	10	5	8	2	4	1	9	7	11	3
$GPC_{7}$	Mean	5.9318E+02	8.0019E+02	2.4750E+02	5.0637E+01	3.6530E+02	2.2852E+02	**7.7402E+01**	-2.860E+00	5.522E+02	9.105E+06	5.0637E+01
	Std	2.9809E+02	1.1585E+02	2.3388E+02	7.5341E+01	1.8888E+02	1.0203E+02	**6.1565E+01**	1.192E+02	3.044E+02	4.528E+06	7.0755E+01
	p-rank	−	−	−	−	−	−	−	−	−	−	
	f-rank	9	10	6	2	7	5	3	4	8	11	1
$GPC_{8}$	Mean	5.7614E+00	6.1826E+00	6.0490E+00	5.6594E+00	5.7615E+00	5.8395E+00	**4.2537E+00**	5.594E+00	5.343E+00	1.219E+09	5.3032E+00
	Std	5.3632E–01	3.6138E–01	3.9446E–01	3.4696E–01	5.2577E–01	3.9440E–01	**5.2491E–01**	7.893E–01	3.227E–01	4.388E+08	4.3006E–01
	p-rank	−	−	−	−	−	−	+	−	−	−	
	f-rank	6	10	9	5	7	8	1	4	3	11	2
$GPC_{9}$	Mean	6.2079E+02	3.0304E+03	**1.2186E+03**	5.3861E+00	2.9154E+00	3.1351E+00	6.2241E+00	1.126E+01	6.544E+01	9.207E+08	2.6235E+00
	Std	4.2195E+02	1.4339E+03	**5.0662E+02**	1.8055E+00	3.07977E–01	2.1050E–01	2.9711E+00	1.932E+01	1.743E+02	1.131E+08	8.1972E–02
	p-rank	−	−	−	−	−	−	−	−	−	−	
	f-rank	8	10	9	4	2	3	5	6	7	11	1
$GPC_{10}$	Mean	2.0280E+01	2.0451E+01	2.0361E+01	2.0371E+01	2.0143E+01	2.0402E+01	**1.8951E+01**	2.037E+01	1.993E+01	1.541E+06	1.9678E+01
	Std	3.3555E–01	9.8487E–02	1.1034E–01	8.9158-02	1.0614E–01	6.9634E–01	**3.0286E+00**	7.670E–02	2.424E+00	7.46E+05	3.0152E+00
	p-rank	−	−	−	−	−	−	+	−	−	−	
	f-rank	5	10	6	8	4	9	1	7	3	11	2
w/l/t		01/09/00	01/09/00	01/09/00	01/09/00	02/08/00	01/09/00	04/06/00	01/09/00	01/09/00	01/09/00	NA
Average f-rank		6.80	8.70	7.80	5.70	4.60	4.80	3.70	5.60	5.30	10.60	2.04
Overall f-rank		8	10	9	7	3	4	2	6	5	11	1

Significant values are in [bold].

### Quantative analysis of GPC algorithm

In this section, we perform a qualitative and quantitative analysis of the GPC algorithm. We are using a set of seven classical benchmark problems^62^, as given in Table 4.

**Table 4:** Classical benchmark functions (*F*1–*F*14).

Func.	Mathematical formulation	Range	Dim.
F1	$f(\textbf{x}) = \sum _{i=1}^{n} x_i^2$	$[-100, 100]^n$	30
F2	$f(\textbf{x}) = \sum _{i=1}^{n} |x_i| + \prod _{i=1}^{n} |x_i|$	$[-10, 10]^n$	30
F3	$f(\textbf{x}) = \max _{i} |x_i|$	$[-100, 100]^n$	30
F4	$f(\textbf{x}) = \sum _{i=1}^{n} \left\lfloor x_i + 0.5 \right\rfloor ^2$	$[-10, 10]^n$	30
F5	$f(\textbf{x}) = \sum _{i=1}^{n} i \cdot x_i^4 + \text {rand}[0,1)$	$[-1.28, 1.28]^n$	30
F6	$f(\textbf{x}) = \frac{1}{4000} \sum x_i^2 - \prod \cos \left( \frac{x_i}{\sqrt{i}}\right) + 1$	$[-600, 600]^n$	30
F7	$f(x_1, x_2) = [1 + (x_1 + x_2 +1)^2(19 - 14x_1 + 3x_1^2 - 14x_2 + 6x_1x_2 + 3x_2^2)]$	$[-2, 2]^2$	2
	$$\times [30 + (2x_1 - 3x_2)^2(18 - 32x_1 + 12x_1^2 + 48x_2 - 36x_1x_2 + 27x_2^2)]$$		

The evaluation of the proposed algorithm uses a set of informative visualization graphs that collectively provide a comprehensive understanding of its search behavior and performance. The exploration–exploitation balance plot tracks how the algorithm transitions from global exploration to local exploitation over the course of iterations. The convergence graph (on a logarithmic fitness scale) illustrates how quickly and effectively the algorithm minimizes the objective function, offering insight into its optimization speed and stability. The fitness distribution plot captures the spread and concentration of fitness values throughout the population, revealing how diversity evolves during the search. Principal Component Analysis (PCA) trajectories visualize the movement of the population in the reduced-dimensional solution space, highlighting patterns in search directionality and convergence. Lastly, the agent-wise fitness plot shows the performance of the individual agents in iterations, indicating how the population collectively approaches optimal solutions.

**Fig. 8:**
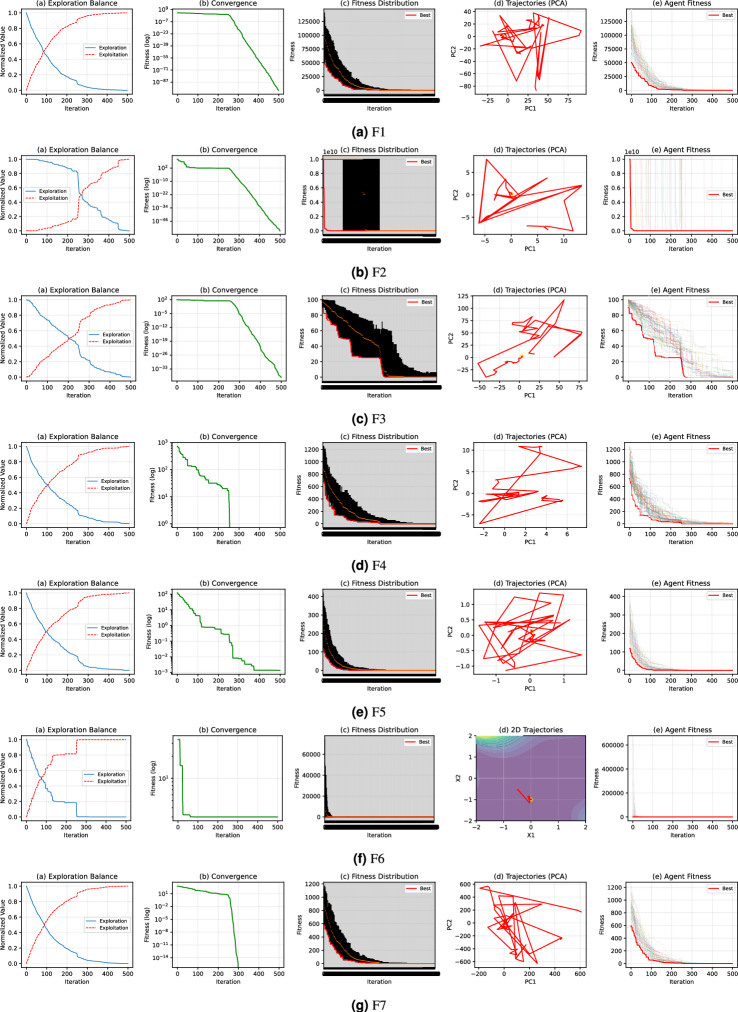
Quantitative analysis of GPC.

From the results in Fig. 8, the proposed GPC algorithm shows robust and adaptive performance. The exploration–exploitation plots confirm that GPC maintains diversity early on and shifts to focused search later, preventing premature convergence. The convergence curves show a consistent reduction in fitness values, suggesting effective optimization over time. The fitness distribution plots reveal that GPC encourages both competition and refinement within the population, with the spread narrowing as better solutions dominate. The PCA trajectories exhibit structured movement toward specific regions in the search space, reflecting guided and non-random exploration. Finally, agent-wise fitness trends indicate population-level improvement and strong convergence toward high-quality solutions. Together, these observations affirm that GPC is well-equipped to handle complex, high-dimensional optimization problems with both efficiency and stability.

### Results on parameter extraction of PEMFC models

In this section, we address the parameter extraction challenges of four distinct PEMFC models utilizing the GPC algorithm to perform a comprehensive performance analysis of the proposed GPC algorithm. The decision to integrate these three algorithms was driven by their complementary strengths in addressing the two-fold challenge inherent in PEMFC parameter estimation: (1) Navigating a complex, high-dimensional and multimodal error surface and (2) achieving precise convergence to the true parameters while avoiding premature stagnation. The parameters that require extraction in the model mentioned above have been listed in Table 5 74li2020accurate^84,85^. In addition, this table clearly represents the upper as well as lower limits for each parameter. The traditional Ballard Mark V, NedStack PS6 PEMFC model, Temasek, and the BCS 500 W PEMFC model, with their datasheets shown in Table 6^25,83–85^. The other Statistical error tests such as SSE (Minimum , mean, and standard deviation (Std)), IAE, MBE, MAE, MSE, and RMSE values for all four PEMFC stacks are presented in Table 7.

To evaluate the performance of the GPC algorithm, several well-recognized MHA are compared, including ZOA^51^, SCHO^52^, PSA^53^, SABO^54^, YDSE^55^, EDO^56^, RIME^57^, CDO^58^, COA^59^, HHO^60^, and GWO^61^. To ensure a fair comparison between GPC and other algorithms, all are subjected to the population size (*P*= 50) and the maximum number of iterations (*T* = 400) with 30 runs.

**Table 5:** Practical upper and lower limits for the parameters’ estimation.

Bound	$\xi _{a}$	$\xi _{b}$	$\xi _{c}$	$\xi _{d}$	$R_{con}\left( \Omega \right)$	$\beta$	$\lambda$
LB	– 1.19969E+00	1.0000E–3	3.6000E–05	– 2.6000E–04	1.0000E–04	1.3600E–02	1.0000E+01
UB	– 8532E–01	5.0000E–03	9.8000E–05	– 9.5400E–05	8.0000E–04	5.0000E–01	2.4000E+01

**Table 6:** Specifications of, Temasek, Ballard_Mark-V, BCS500-W as well as NedStack PS6.

	Ballard_Mark-V	BCS500-W	NedStack PS6	Temasek
N	35	32	65	20
$J_{max}$	1.5	0.469	5	1.5
$P_{H2} (bar)$	1	1	1	0.5
l ($\mu$m)	178	178	178	51
A $(cm^2)$	50.6	64	240	150
T (K)	343	333	343.15	323
$P_{O2} (bar)$	1	0.2075	1	0.5

**Table 7:** Statistical error tests for all four PEMFC stack.

S.no	Algorithm	Objective function			IAE	MBE	MAE	MSE	RMSE
		Minimum	Mean	Std					
CASE I : NedStack PS6									
1	GPC	2.267687	2.281716	0.012378	6.411737	0.00577	0.213725	0.07559	0.274936
2	ZOA	2.268365	2.370253	0.092782	6.393405	– 0.00013	0.213114	0.075612	0.274977
3	SCHO	2.447831	3.366872	0.588855	6.713101	– 0.00712	0.22377	0.081594	0.285647
4	PSA	2.349586	4.20473	3.007886	6.781009	0.039266	0.226034	0.07832	0.279856
5	SABO	2.95804	3.696988	0.410863	7.380344	0.011761	0.246011	0.098601	0.314008
6	YDSE	2.403032	2.550963	0.112013	6.499391	– 0.01088	0.216646	0.080101	0.283021
7	EDO	2.50108	2.90447	0.235885	6.848847	0.000606	0.228295	0.083369	0.288737
8	RIME	2.285971	2.503289	0.264613	6.416289	– 0.00027	0.213876	0.076199	0.276042
9	CDO	2.496433	3.197543	0.394407	7.10536	0.067296	0.236845	0.083214	0.288469
10	COA	2.482398	16.329	17.96253	6.626224	0.012427	0.220874	0.082747	0.287657
11	HHO	2.330158	4.1509	1.431906	6.485056	– 8.3E–05	0.216169	0.077672	0.278697
12	GWO	2.301123	3.048242	0.594742	6.474575	0.004843	0.215819	0.076704	0.276955
CASE II :Ballard Mark V									
1	GPC	0.813912	0.813919	1.19E–05	2.509658	3.3E–05	0.193051	0.062609	0.250217
2	ZOA	0.823312	0.862624	0.023348	2.52628	8.01E–06	0.194329	0.063332	0.251658
3	SCHO	0.819595	0.890729	0.073262	2.548133	– 0.01071	0.19601	0.063046	0.251089
4	PSA	0.836537	1.305572	0.346251	2.494412	– 0.00838	0.191878	0.064349	0.253671
5	SABO	0.953818	2.202805	1.673595	2.708742	0.01243	0.208365	0.073371	0.27087
6	YDSE	0.813939	0.814728	0.000554	2.506742	0.000483	0.192826	0.062611	0.250221
7	EDO	0.81439	0.827771	0.007198	2.508804	– 0.00159	0.192985	0.062645	0.250291
8	RIME	0.813979	0.875387	0.076183	2.506412	0.001574	0.192801	0.062614	0.250227
9	CDO	1.781255	1.917044	0.13879	3.707171	– 0.03599	0.285167	0.137267	0.370495
10	COA	0.838759	7.469678	12.59914	2.484699	– 0.00669	0.191131	0.06452	0.254008
11	HHO	0.909931	2.171336	1.100664	2.652076	– 0.00022	0.204006	0.069995	0.264565
12	GWO	0.814886	0.85447	0.032931	2.505479	0.002686	0.192729	0.062649	0.250298
CASE III :BCS 500 W									
1	GPC	0.011699	0.011702	2.8E–06	0.234845	– 0.00018	0.013047	0.00065	0.025494
2	ZOA	0.012497	0.023294	0.006145	0.273599	0.000181	0.0152	0.000694	0.026349
3	SCHO	0.012752	0.025285	0.009964	0.306834	– 0.00524	0.017046	0.000708	0.026616
4	PSA	0.027469	0.286863	0.51951	0.507265	0.000444	0.028181	0.001526	0.039064
5	SABO	0.044445	0.349763	0.29687	0.696977	0.028717	0.038721	0.002469	0.049691
6	YDSE	0.011704	0.011786	5.1E–05	0.234856	– 6.5E–05	0.013048	0.00065	0.025499
7	EDO	0.011908	0.013582	0.001319	0.212715	1.46E–05	0.011818	0.000662	0.025721
8	RIME	0.012326	0.019906	0.006801	0.219671	– 0.0003	0.012204	0.000685	0.026168
9	CDO	1.594396	4.382463	0.534277	4.303728	0.239096	0.239096	0.088578	0.29762
10	COA	0.027964	2.199596	1.802482	0.495684	0.005031	0.027538	0.001554	0.039415
11	HHO	0.028383	2.546672	1.998167	0.533353	0.000457	0.029631	0.001577	0.039709
12	GWO	0.01197	0.015778	0.003935	0.21046	0.001616	0.011692	0.000665	0.025788
CASE IV :Temasek Stack									
1	GPC	0.123277	0.123338	8.08E–05	1.123446	– 1.1E–06	0.080246	0.008805	0.093838
2	ZOA	0.123282	0.129403	0.005534	1.124669	4.49E–05	0.080333	0.008806	0.09384
3	SCHO	0.124343	0.147219	0.017405	1.13627	0.002734	0.081162	0.008882	0.094242
4	PSA	0.123313	0.208102	0.123007	1.124563	– 0.00093	0.080326	0.008808	0.093851
5	SABO	0.127516	0.140673	0.017099	1.164312	0.011565	0.083165	0.009108	0.095437
6	YDSE	0.123291	0.124988	0.002193	1.120813	– 0.00047	0.080058	0.008807	0.093843
7	EDO	0.123691	0.126943	0.002715	1.139366	0.004234	0.081383	0.008835	0.093995
8	RIME	0.123277	0.12823	0.007493	1.123072	– 0.00015	0.080219	0.008806	0.093838
9	CDO	0.124345	0.13533	0.006247	1.148625	0.004698	0.082045	0.008882	0.094243
10	COA	0.123311	0.155337	0.043357	1.121898	– 0.00147	0.080136	0.008808	0.093851
11	HHO	0.127156	0.163503	0.062332	1.139663	8.05E–05	0.081404	0.009083	0.095303
12	GWO	0.124193	0.134406	0.009724	1.130645	– 0.00013	0.08076	0.008871	0.094186

#### NedStack PS6 PEMFC model

**Fig. 9:**
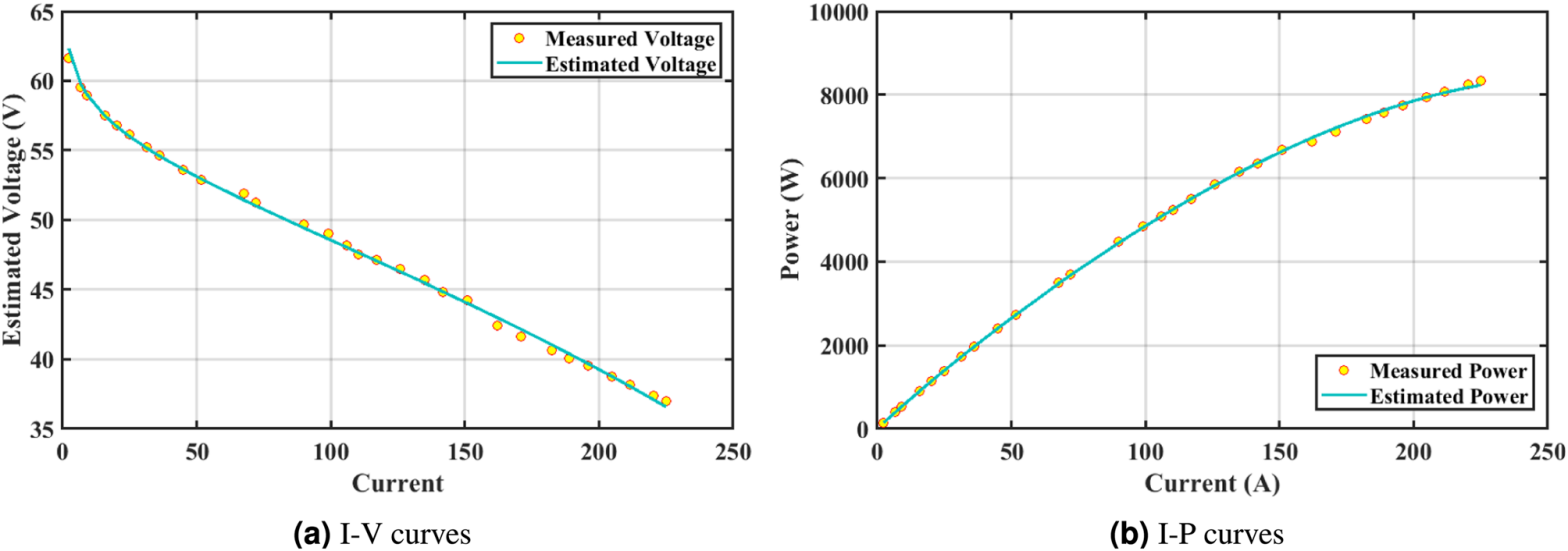
Model curves of NedStack PS6 PEMFC stack.

The effectiveness of the GPC algorithm is demonstrated using a commonly highlighted PEMFC (NedSstack PS6) in existing literature, with a rated power of 6kW. The data specifications and upper and lower limits are presented in Tables 6 and 5. The experimental Power ($P_{exper}$), experimental voltage ($V_{exper}$), estimated voltage ($V_{estimat}$), model-estimated power ($P_{estimat}$), and IAE values obtained by the GPC algorithm for NedStack PS6 PEMFC 8. The optimal parameter outcomes determined by different algorithms for the NedSstack PS6 stack are presented in Table 9. Table 9 show that when considering the same function evaluations, certain methods, such as ZOA^51^, SCHO^52^, PSA^53^, SABO^54^, YDSE^55^, EDO^56^, RIME^57^, CDO^58^, COA^59^, HHO^60^, and GWO^61^, achieve a range of optimal SSE values. However, the GPC algorithm obtains the lowest SSE value (2.26768E + 00) for the NedSstack PS6 PEMFC stacks. This gives further confirmation that the parameter values obtained by the proposed GPC algorithm are very precise and reliable. Figure 9a clearly shows that the model curves (IV) closely align with the experimental data for NedStack PS6, and there is little variation between them.

**Fig. 10:**
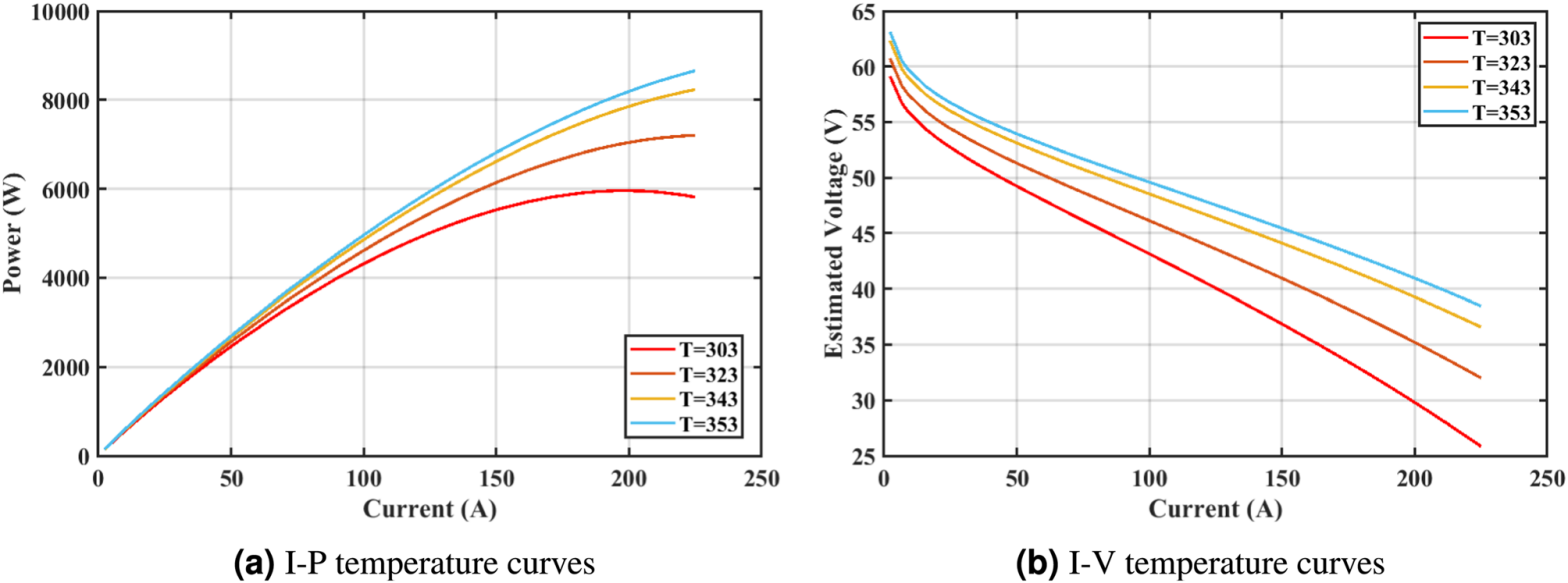
NedStack PS6 stack performance plots based on GPC algorithm parameters extraction under different operating conditions.

**Fig. 11:**
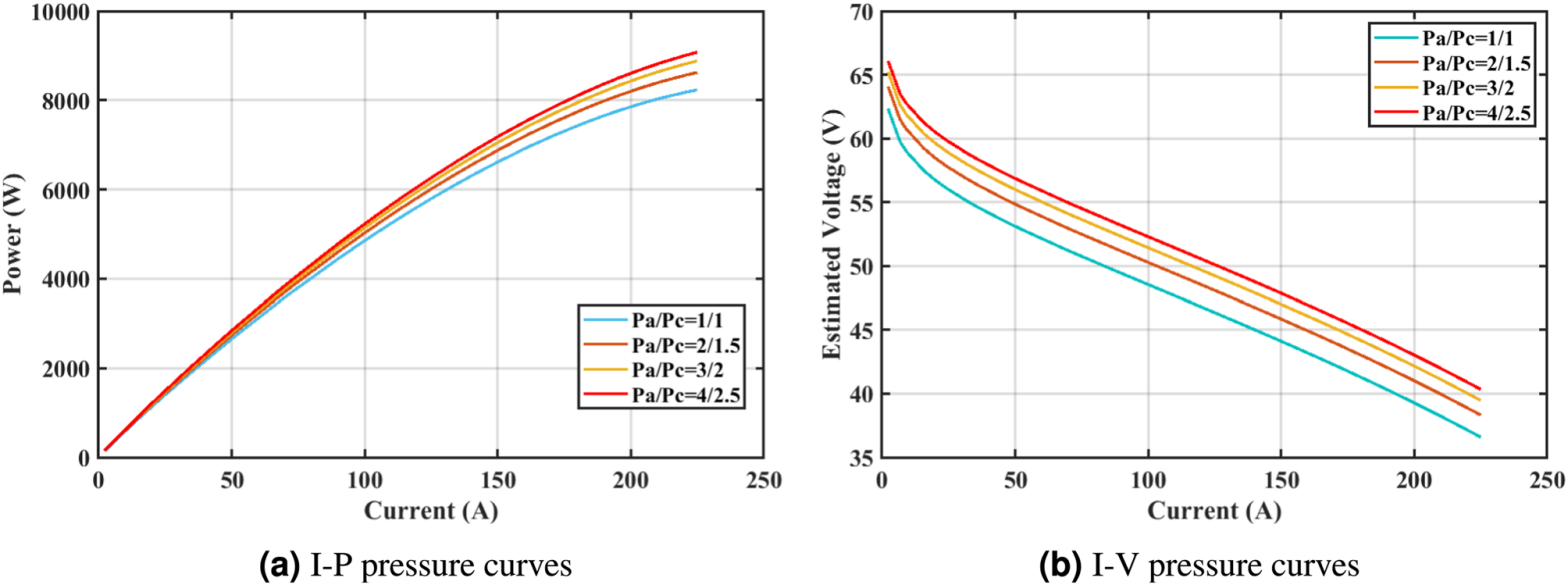
NedStack PS6 stack performance plots based on GPC algorithm parameters extraction under different operating conditions.

**Fig. 12:**
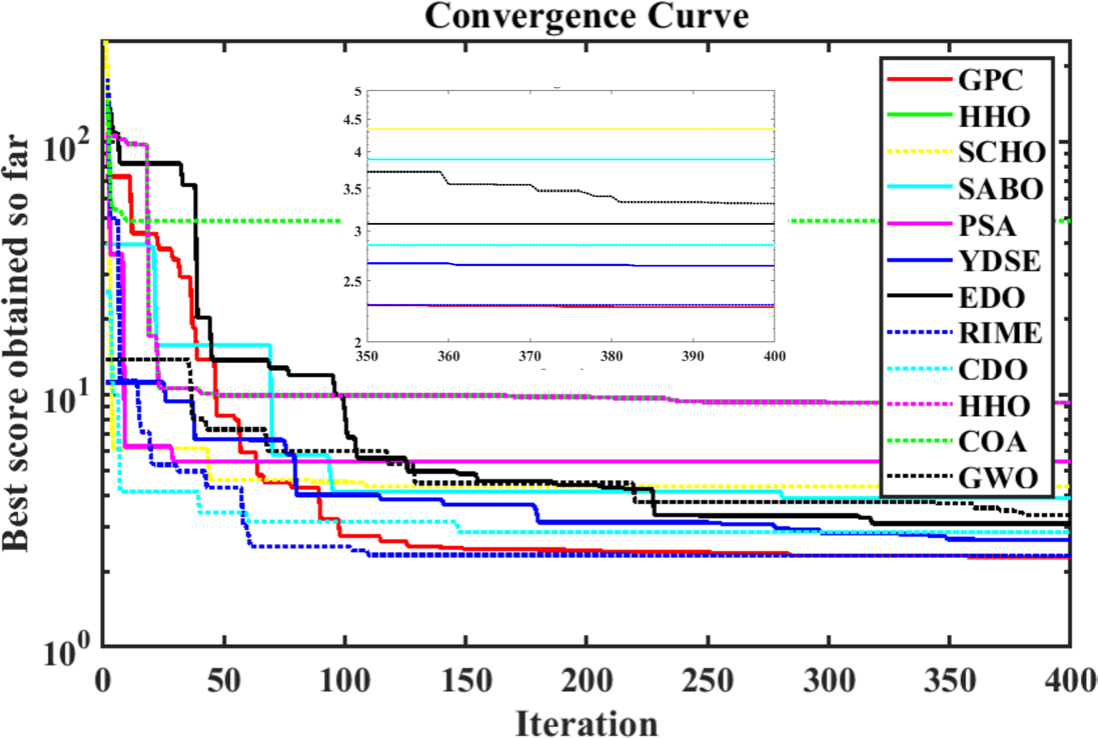
The convergence curves obtained from 400 iterations and 30 runs of 12 algorithms utilized to NedStackPS6 PEMFC stack parameter extraction.

**Fig. 13:**
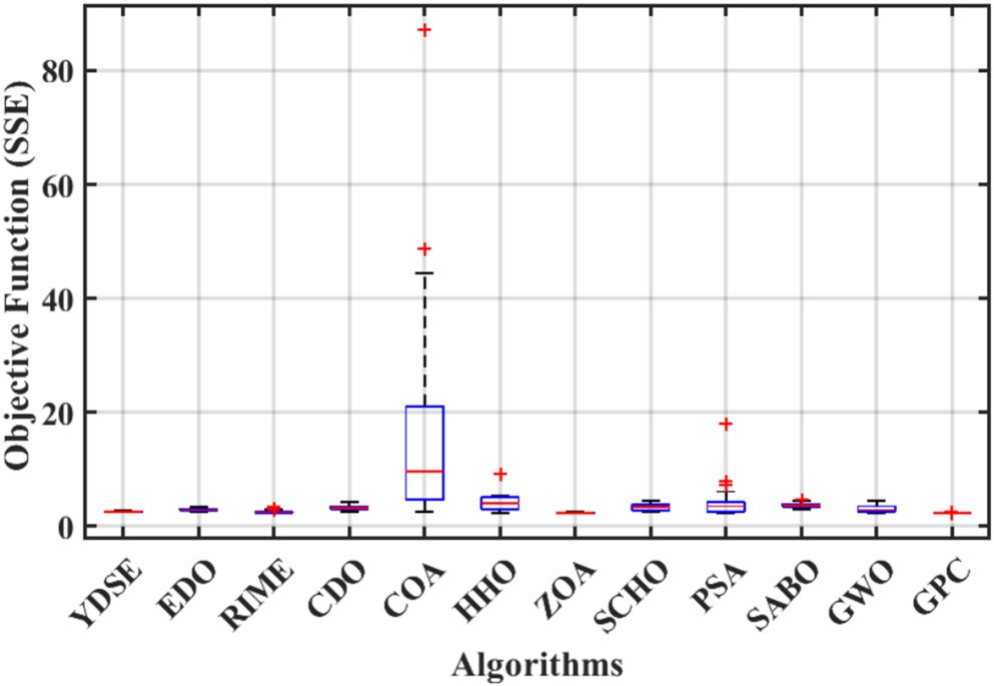
The boxplot curves obtained 30 runs of 12 algorithms utilized to NedStackPS6 PEMFC stack parameter extraction.

Furthermore, the curves of the PEMFC model (PI) are shown in Fig. 9b, which provides additional evidence that the GPC algorithm is accurate in analyzing the parameters of the NedStack PS6 PEMFC. Temperature variations are simulated at four different temperatures: 303, 323, 343, and 353K are presented in Fig. 10a and b for the I-P and I-V curves, respectively. These simulations are carried out under constant partial pressures (that is, $P_{H2}$ / $P_{O2}$ = 1.000 / 1.000 (bar)). It has been observed that as the temperature rises, there is an increase in the output voltage of the stack. Figure 11a and b illustrate the model curves stated in terms of the I-P and I-V curves. Initially, the I-P and I-V curves are graphed at pressures ($P_{H2}$/ $P_{O2}$) of 1.000/1.000, 2.000/1.500, 3.000/2.000 and 4.000/2.500 bar, respectively. These measurements were taken at a constant stack temperature of 343 K and are shown in Figure 11a and b respectively. When the supply pressures of the ($P_{H2}$/ $P_{O2}$) increase, there is an observed enhancement in the output voltage of the stack. Using a similar simulation environment, Figure 12, illustrates the average convergence curves of 400 iterations of the proposed GPC, ZOA, SCHO, PSA, SABO, YDSE, EDO, RIME, CDO, COA, HHO, and GWO for the NedStack PS6 PEMFCs stack. Figure 13 shows the ranges of the final objective function values after 30 runs of the GPC, ZOA, SCHO, PSA, SABO, YDSE, EDO, RIME, CDO, COA, HHO, and GWO algorithms for the extraction of parameters from the NedStack PS6 PEMFCs stack. Based on the size of the box and the number of outliers, it is evident from Fig. 13 that the proposed GPC algorithm outperforms the other 11 algorithms. The results of the Friedman ranking test are given in Table 10. The Friedman test assesses algorithms based on their overall performance, with GPC achieving the highest average rank of 2.103926. Table 10, clearly indicates that the GPC algorithm has achieved the highest ranking ($I^{st}$ rank). The Wilcoxon ranking test results indicate that GPC significantly outperforms the others, evidenced by 465 winner, no losses, and minimal p-values ranging from 2.03E–07 to 3.02E–11. The Friedman and Wilcoxon ranking test clearly demonstrates that the GPC algorithm is superior in terms of precision as well as accuracy compared to the MH algorithms.

**Table 8:** The $V_{estimat}$, $P_{estimat}$, and IAE values obtained by the GPC algorithm for NedStackPS6 PEMFC.

S.no	$V_{exper}$	$V_{estimat}$	$I_{exper}$	$V_{exper}$- $V_{estimat}$	$\big| V_{exper}-$ $V_{estimat} \big|$	$P_{exper}$	$P_{estimat}$	$P_{exper}$- $P_{estimat}$	$\big| P_{exper}-$ $P_{estimat} \big|$
1	61.64	62.3281	2.25	– 0.6881	0.688102	138.69	140.2382	– 1.54823	1.548229
2	59.57	59.75513	6.75	– 0.18513	0.185129	402.0975	403.3471	– 1.24962	1.24962
3	58.94	59.02428	9	– 0.08428	0.084285	530.46	531.2186	– 0.75856	0.758561
4	57.54	57.47379	15.75	0.066206	0.066206	906.255	905.2123	1.042741	1.042741
5	56.8	56.69627	20.25	0.103726	0.103726	1150.2	1148.1	2.100447	2.100447
6	56.13	56.02413	24.75	0.105865	0.105865	1389.218	1386.597	2.620163	2.620163
7	55.23	55.13871	31.5	0.091295	0.091295	1739.745	1736.869	2.875788	2.875788
8	54.66	54.60327	36	0.056729	0.056729	1967.76	1965.718	2.042253	2.042253
9	53.61	53.6181	45	– 0.0081	0.008097	2412.45	2412.814	– 0.36437	0.364373
10	52.86	52.93088	51.75	– 0.07088	0.070881	2735.505	2739.173	– 3.66807	3.66807
11	51.91	51.43086	67.5	0.479142	0.479142	3503.925	3471.583	32.34209	32.34209
12	51.22	51.01968	72	0.200325	0.200325	3687.84	3673.417	14.42337	14.42337
13	49.66	49.4166	90	0.243396	0.243396	4469.4	4447.494	21.90564	21.90564
14	49	48.62858	99	0.371421	0.371421	4851	4814.229	36.77069	36.77069
15	48.15	48.03503	105.8	0.114974	0.114974	5094.27	5082.106	12.16425	12.16425
16	47.52	47.64218	110.3	– 0.12218	0.122181	5241.456	5254.933	– 13.4766	13.4766
17	47.1	47.05614	117	0.043856	0.043856	5510.7	5505.569	5.131208	5.131208
18	46.48	46.26479	126	0.215209	0.215209	5856.48	5829.364	27.11634	27.11634
19	45.66	45.46616	135	0.193838	0.193838	6164.1	6137.932	26.16817	26.16817
20	44.85	44.85639	141.8	– 0.00639	0.006386	6359.73	6360.635	– 0.9055	0.905498
21	44.24	44.03901	150.8	0.20099	0.20099	6671.392	6641.083	30.30932	30.30932
22	42.45	43.00232	162	– 0.55232	0.552319	6876.9	6966.376	– 89.4758	89.47576
23	41.66	42.151	171	– 0.491	0.491001	7123.86	7207.821	– 83.9611	83.96112
24	40.68	41.05565	182.3	– 0.37565	0.375646	7415.964	7484.444	– 68.4803	68.48027
25	40.09	40.39074	189	– 0.30074	0.300735	7577.01	7633.849	– 56.8389	56.83895
26	39.51	39.70306	195.8	– 0.19306	0.193063	7736.058	7773.86	– 37.8018	37.80178
27	38.73	38.7715	204.8	– 0.0415	0.041498	7931.904	7940.403	– 8.49872	8.49872
28	38.15	38.06101	211.5	0.088986	0.088986	8068.725	8049.904	18.82062	18.82062
29	37.38	37.08209	220.5	0.297911	0.297911	8242.29	8176.601	65.68927	65.68927
30	37	36.58145	225	0.418546	0.418546	8325	8230.827	94.17276	94.17276
IAE (V)				6.411737	IAE (P)			762.7227	

**Table 9:** Parameter estimation and statistical measures comparison of various MH algorithms for NedStackPS6 PEMFC.

S.no	Algorithms	Objective function	$\xi _{a}$	$\xi _{b}$	$\xi _{c}$	$\xi _{d}$	$R_{con}\left( \Omega \right)$	$\beta$	$\lambda$
1	GPC	2.267687	– 0.98488	0.003634	9.7E–05	– 9.5E–05	0.0001	0.0136	12.63762
2	ZOA	2.268365	– 0.85372	0.002416	3.72E–05	– 9.5E–05	0.000101	0.014562	12.65956
3	SCHO	2.447831	– 1.1748	0.004054	8.73E–05	– 9.6E–05	0.000104	0.043096	13.08828
4	PSA	2.349586	– 0.97677	0.002754	0.000036	– 9.5E–05	0.0001	0.0136	12.71295
5	SABO	2.95804	– 0.8532	0.002551	4.65E–05	– 9.5E–05	0.00021	0.113416	15.18183
6	YDSE	2.403032	– 0.88439	0.003159	8.36E–05	– 9.5E–05	0.000153	0.030912	13.3186
7	EDO	2.50108	– 1.18621	0.003575	5.07E–05	– 9.6E–05	0.000154	0.0136	13.31647
8	RIME	2.285971	– 0.99467	0.003116	5.78E–05	– 9.5E–05	0.000116	0.0136	12.79751
9	CDO	2.496433	– 0.8532	0.002396	0.000036	– 9.5E–05	0.000104	0.077835	13.31888
10	COA	2.482398	– 0.8532	0.003002	7.88E–05	– 9.5E–05	0.0001	0.066014	12.96025
11	HHO	2.330158	– 0.8532	0.002555	4.71E–05	– 9.5E–05	0.000134	0.021973	13.05329
12	GWO	2.301123	– 0.95114	0.002844	4.74E–05	– 9.5E–05	0.00011	0.034966	12.93686
13	GSA^86^	2.58	– 0.874	0.0033487	8.93E–05	– 9.54E–05	0.0002388	0.0565881	18.8
14	MRFO^25,87^	2.88702	– 1.05602	0.00313	4.61E–05	– 9.58E–05	0.000166	0.0547	20.188
15	SSA^43^	2.5711	−0.989	0.00333	7.41E–05	– 9.54E–05	0.000256	0.0426	20.5
16	VSA^27^	2.34	– 0.895	0.00335	9.75E–05	– 9.54E–05	0.000103	0.0429	13.0
17	GA^88^	2.41	– 1.1997	0.003417	3.6E–05	– 9.54E–05	0.0001376	0.0359	13.00
18	PSO^33^	4.050	– 0.8532	0.002604	4.9E–05	– 9.54E–05	0.0001396	0.5	23.00

**Table 10:** Friedman and Wilcoxon rank test of various MH algorithms for NedStackPS6 PEMFC.

S.No	Algorithm	Friedman’s rank,	Rank	Winner	Loser	Wilcoxon’s p value
1	GPC	2.103926	1			
2	ZOA	2.911832	2	465.00	0	2.0300E–07
3	SCHO	8.513548	7	465.00	0	3.0200E–11
4	PSA	8.857507	9	465.00	0	3.02E–11
5	SABO	10.23156	11	465.00	0	3.0200E–11
6	YDSE	5.114724	4	465.00	0	3.0200E–11
7	EDO	7.539125	6	465	0	3.02E–11
8	RIME	3.593653	3	465.00	0	3.4700E–10
9	CDO	8.546709	8	465.00	0	3.0200E–11
10	COA	11.83236	12	465.00	0	3.0200E–11
11	HHO	9.230874	9	465.00	0	3.0200E–11
12	GWO	6.890946	5	465.00	0	4.9800E–11

#### Ballard Mark V

**Fig. 14:**
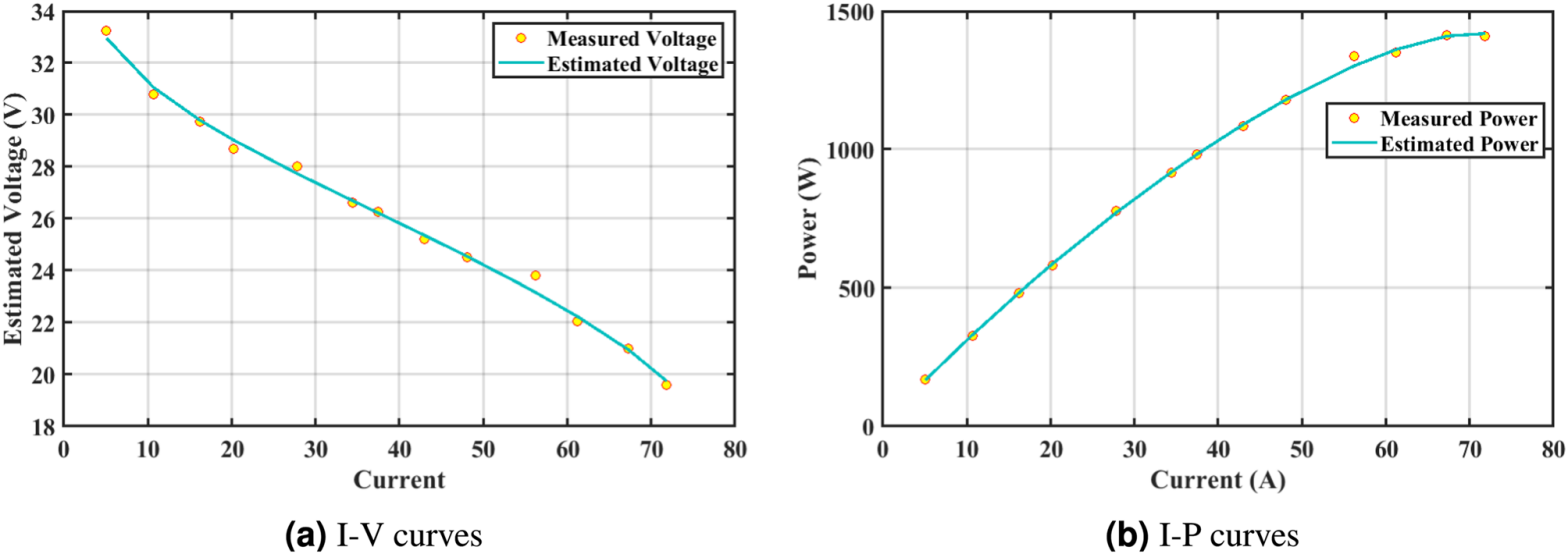
Model curves of Ballard Mark V PEMFC stack.

The Ballard mark V PEMFC stack comprises 35 individual cells that are connected in series with a membrane thickness of 178 $\mu$m. The upper and lower limits and data specifications and are presented in Tables 5 and 6. The experimental Power ($P_{exper}$), experimental voltage ($V_{exper}$), estimated voltage ($V_{estimat}$), model-estimated power ($P_{estimat}$), and IAE values obtained by the GPC algorithm for Ballard Mark V PEMFC 11. The GPC, ZOA, SCHO, PSA, SABO, YDSE, EDO, RIME, CDO, COA, HHO, and GWO, MH optimization algorithms have been utilized in order to achieve optimal parameter extraction for this model. The resulting values, which have been found to be the best according to the SSE objective, have been organized and presented in Table 15. From this table, the GPC shows superior performance along with the lowest objective function (SSE) value of 0.813912 compared to other MH algorithms. Figure 14a and b clearly demonstrate that the model curves (I-V as well as I-P curves) closely align with the experimental data for the Ballard Mark V PEMFC stack and there is little variation between them. This gives further confirmation that the parameter values obtained by the proposed GPC algorithm are very precise and reliable.

**Table 11:** The $V_{estimat}$, $P_{estimat}$, and IAE values obtained by the GPC algorithm for Ballard Mark V PEMFC.

S No.	$V_{exper}$	$V_{estimat}$	$I_{exper}$	$V_{exper}$- $V_{estimat}$	$\big| V_{exper}-$ $V_{estimat} \big|$	$P_{exper}$	$P_{estimat}$	$P_{exper}$- $P_{estimat}$	$\big| P_{exper}-$ $P_{estimat} \big|$
1	33.25	32.96755	5.06	0.28245	0.28245	168.245	166.8158	1.429197	1.429197
2	30.8	31.06831	10.626	– 0.26831	0.268313	327.2808	330.1319	– 2.85109	2.851089
3	29.75	29.79399	16.192	– 0.04399	0.043986	481.712	482.4242	– 0.71222	0.71222
4	28.7	29.02021	20.24	– 0.32021	0.320209	580.888	587.369	– 6.48102	6.481024
5	28	27.73154	27.83	0.268463	0.268463	779.24	771.7687	7.471328	7.471328
6	26.6	26.69303	34.408	– 0.09303	0.093029	915.2528	918.4537	– 3.20095	3.200947
7	26.25	26.2217	37.444	0.028304	0.028304	982.905	981.8452	1.059801	1.059801
8	25.2	25.35378	43.01	– 0.15378	0.153775	1083.852	1090.466	– 6.61387	6.613871
9	24.5	24.54563	48.07	– 0.04563	0.045634	1177.715	1179.909	– 2.19362	2.193617
10	23.8	23.17304	56.166	0.62696	0.62696	1336.751	1301.537	35.21383	35.21383
11	22.05	22.23229	61.226	– 0.18229	0.182285	1350.033	1361.194	– 11.1606	11.1606
12	21	20.95113	67.298	0.048867	0.048867	1413.258	1409.969	3.288636	3.288636
13	19.6	19.74738	71.852	– 0.14738	0.147384	1408.299	1418.889	– 10.5898	10.58983
IAE (V)				2.509658	IAE (P)			92.26599	

**Fig. 15:**
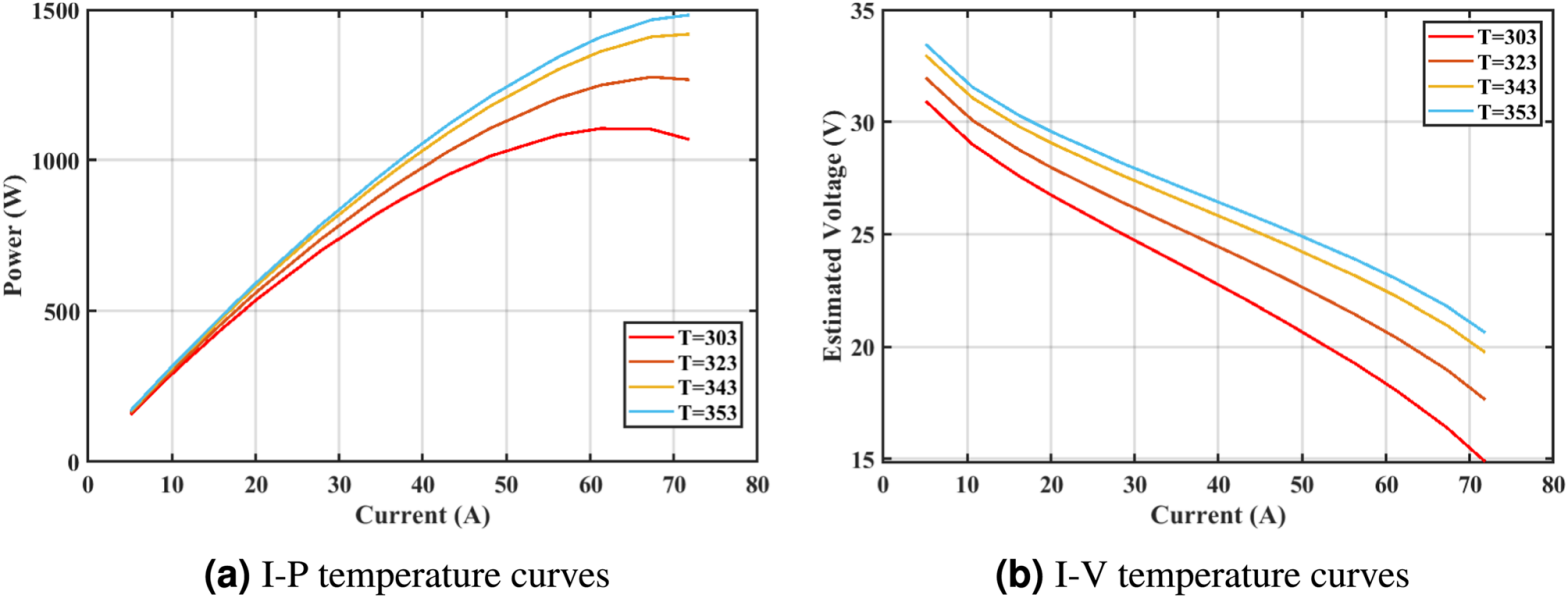
Ballard Mark V stack performance plots based on GPC algorithm parameters extraction under different operating conditions.

**Fig. 16:**
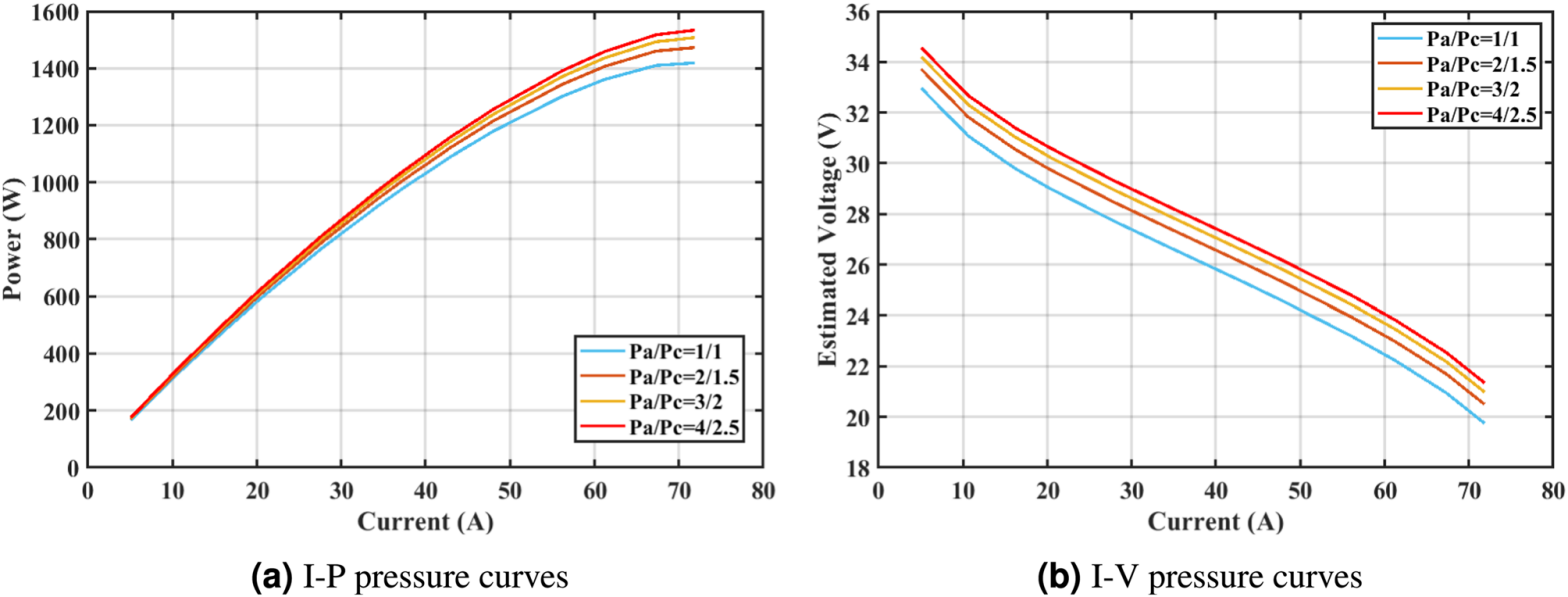
Ballard Mark V stack performance plots based on GPC algorithm parameters extraction under different operating conditions.

**Fig. 17:**
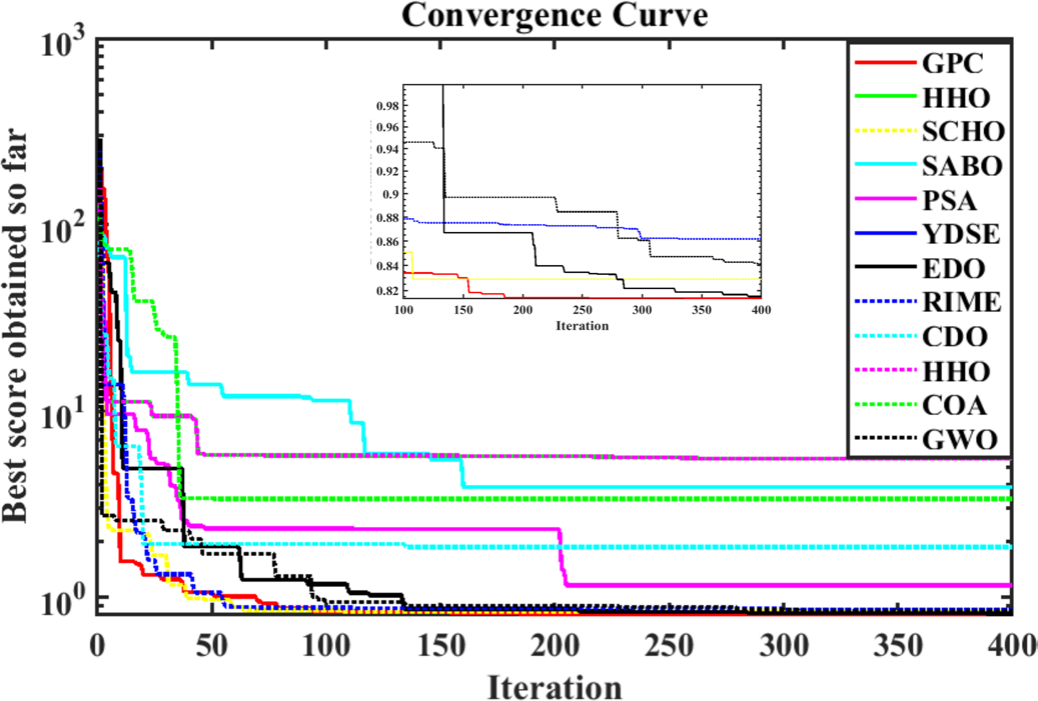
The convergence curves obtained from 400 iterations and 30 runs of 12 algorithms utilized to Ballard Mark V PEMFC stack parameter extraction.

**Fig. 18:**
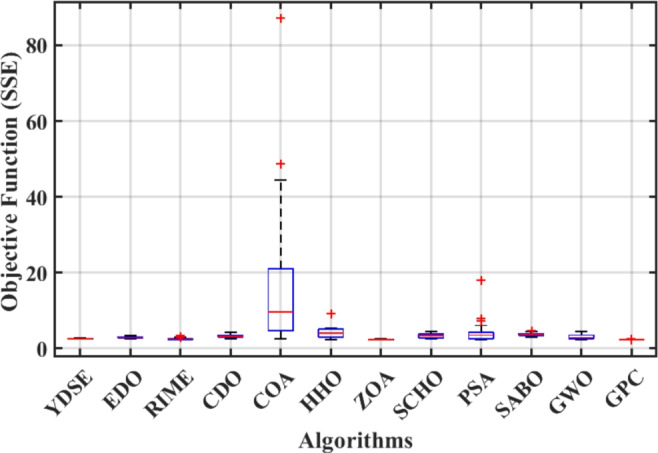
The boxplot curves obtained from 30 runs of 12 algorithms utilized to Ballard Mark V PEMFC stack parameter extraction.

Furthermore, the temperature variations are simulated at four different temperatures: 303, 323, 343, and 353K with constant partial pressures (that is, $P_{H2}$ / $P_{O2}$ = 1.000 / 1.000 (bar)) are shown in Fig. 15a and b for the I-P and I-V curves, respectively Table 12. It has been observed that as the temperature rises, there is an increase in the output voltage of the stack. The pressure variations are then simulated at four different temperatures: ($P_{H2}$/ $P_{O2}$) of 1.000/1.000, 2.000/1.500, 3.000/2.000 and 4.000/2.500 bar with constant temperature (ie 343K) are shown in Fig. 16 (16a and b) for I-P and I-V curves, respectively. When the supply pressures of the ($P_{H2}$/ $P_{O2}$) increase, an enhancement is observed in the output voltage of the stack. Using the same simulation environment, Fig. 17, shows the convergence curves obtained from 400 iterations and 30 runs of 12 algorithms (GPC, ZOA, SCHO, PSA, SABO, YDSE, EDO, RIME, CDO, COA, HHO and GWO) used to extract the parameters of the Ballard Mark V PEMFC stack. Figure 18 illustrates the box plot curves obtained from 30 runs of the GPC, ZOA, SCHO, PSA, SABO, YDSE, EDO, RIME, CDO, COA, HHO and GWO algorithms utilized to extract the parameters from the Ballard Mark V PEMFC stack. Based on the size of the box and the number of outliers, it is evident from Fig. 18 that the proposed GPC algorithm outperforms the other 11 algorithms. Table 13 presents the Friedman and Wilcoxon rank test of various MH algorithms for the Ballard Mark V PEMFC stack. The Friedman test assesses algorithms based on their overall performance, with GPC achieving the highest average rank of 1.856791. From Table 13, it is clearly observed that the GPC algorithm has achieved the highest rank ($I^{st}$). The Wilcoxon ranking test results indicate that GPC significantly outperforms the others, evidenced by 465 winner, no losses, and minimal p-values 3.02E–11. The Friedman and Wilcoxon ranking test clearly demonstrates that the GPC algorithm is superior in terms of precision and accuracy compared to the MH algorithms.

**Table 12:** Parameter estimation and statistical measures comparison of various MH algorithms for Ballard Mark V PEMFC.

S.no	Algorithms	Objective function	$\xi _{a}$	$\xi _{b}$	$\xi _{c}$	$\xi _{d}$	$R_{con}\left( \Omega \right)$	$\beta$	$\lambda$
1	GPC	0.813912	– 1.01123	0.003341	5.85E–05	– 0.00017	0.0001	0.015885	24.000
2	ZOA	0.823312	– 0.86073	0.002658	4.12E–05	– 0.00017	0.000128	0.015728	23.99993
3	SCHO	0.819595	– 1.19969	0.003604	3.81E–05	– 0.00017	0.0001	0.015343	23.9519
4	PSA	0.836537	– 1.19969	0.004007	6.56E–05	– 0.00017	0.0001	0.0136	24.000
5	SABO	0.953818	– 1.17202	0.003601	4.69E–05	– 0.00015	0.000272	0.017743	24.000
6	YDSE	0.813939	– 1.00656	0.00367	8.29E–05	– 0.00017	0.0001	0.015812	24.000
7	EDO	0.81439	– 1.05739	0.00391	8.93E–05	– 0.00017	0.0001	0.015828	24
8	RIME	0.813979	– 1.19227	0.003697	4.62E–05	– 0.00017	0.0001	0.015978	24.000
9	CDO	1.781255	– 0.8532	0.002408	0.000036	– 9.5E–05	0.0008	0.0136	22.96162
10	COA	0.838759	– 1.19785	0.003592	3.6E–05	– 0.00017	0.000101	0.0138	23.94841
11	HHO	0.909931	– 1.19965	0.003674	4.47E–05	– 0.00015	0.000351	0.013687	23.99544
12	GWO	0.814886	– 1.18875	0.003572	3.81E–05	– 0.00017	0.000102	0.016036	24.000
13	NNO^89^	0.85361	– 0.97997	0.003694	9.08710E–05	– 1.62820E–04	0.0001	0.0136	23.000
14	STSA^32^	0.85361	– 0.8532	0.00255805	3.60438E–05	– 1.62828	0.0001	0.0136	23.000
15	ABC– DE^38^	0.853607	– 1.19561	0.00421	8.34036E–05	– 1.62830E–04	0.0001	0.0136	23.0000
16	LSA^21^	0.8140	−1.0624	3.597E–03	6.653E–05	– 16.492E–05	0.0001	0.0188	23.000
17	MRFO^25^	0.8533	– 1.19561	4.2188E–03	8.340E–05	– 1.6280E–04	0.0001	0.0136	23.000
18	DO^34^	0.8292	– 0.8532	2.869E–03	5.933E–05	– 14.75E–05	0.0001	0.0343	23.00

**Table 13:** Friedman and Wilcoxon rank test of various MH algorithms for Ballard Mark V PEMFC.

S.No	Algo	Friedman’s rank	Rank	Winner	Loser	Wilcoxon’s p value
1	GPC	1.856791	1			
2	ZOA	6.406046	7	465.00	0	3.0200E–11
3	SCHO	5.9985	6	465.00	0	3.0200E–11
4	PSA	8.34359	8	465.00	0	3.0200E–11
5	SABO	10.04617	9	465.00	0	3.0200E–11
6	YDSE	2.878735	2	465.00	0	3.6900E–11
7	EDO	4.524407	3	465.00	0	3.0200E–11
8	RIME	5.743132	5	465.00	0	3.0200E–11
9	CDO	10.89223	12	465.00	0	3.0200E–11
10	COA	11.05548	10	465.00	0	3.0200E–11
11	HHO	10.60452	11	465.00	0	3.0200E–11
12	GWO	5.030465	4	465.00	0	3.0200E–11

#### BCS 500 W

**Fig. 19:**
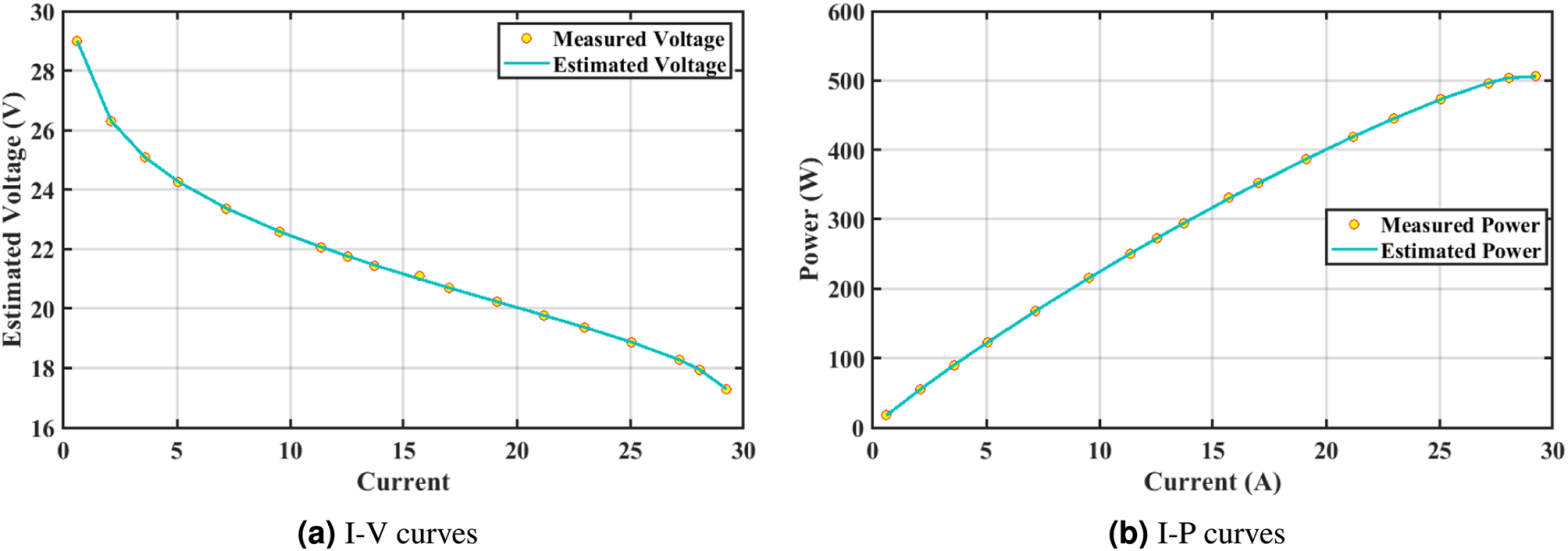
Model curves of BCS500-W PEMFC stack.

The BCS 500-W PEMFC stack operates at a power output of 500 watts as well as current of 30 amperes. The specifications of the BCS 500-W PEMFC stack are presented in Table 6 and can be found in^90,91^. The experimental Power ($P_{exper}$), experimental voltage ($V_{exper}$), the estimated voltage ($V_{estimat}$), model-estimated power ($P_{estimat}$), and IAE values obtained by the GPC algorithm for the BCS500-W PEMFC 14. In addition, Table ?? presents the optimal values of the unknown parameters of the BCS 500-W PEMFC stack obtained by the GPC algorithm and compared to the other MH optimization algorithm. Also, the GPC algorithm obtained the lowest objective function (SSE) value (0.011699). This clearly shows its significant superiority compared to the other MH optimization algorithms reported in the literature. This illustrates that an accurate representation of the BCS 500-W PEMFC stack has been achieved. Figure 19a and b clearly illustrate that the model curves (I-V and I-P curves) closely align with the experimental data for the BCS500-W PEMFC stack, and there is little variation between them. The convergence curves have been obtained from 400 iterations and 30 runs of 12 algorithms utilized to extract parameter values from the BCS500-W PEMFC stack. Figure 22 illustrates the convergence curve of the objective function. Here, it is clear that the convergence curve is continuous and rapidly reaches its final value.

**Table 14:** The $V_{estimat}$, $P_{estimat}$, and IAE values obtained by the GPC algorithm for BCS500-W PEMFC.

SNo.	$V_{exper}$	$V_{estimat}$	$I_{exper}$	$V_{exper}$- $V_{estimat}$	$\big| V_{exper}-$ $V_{estimat} \big|$	$P_{exper}$	$P_{estimat}$	$P_{exper}$- $P_{estimat}$	$\big| P_{exper}-$ $P_{estimat} \big|$
1	29	28.99747	0.6	0.00253	0.00253	17.4	17.39848	0.001518	0.001518
2	26.31	26.30615	2.1	0.00385	0.00385	55.251	55.24291	0.008086	0.008086
3	25.09	25.09376	3.58	– 0.00376	0.003763	89.8222	89.83567	– 0.01347	0.013472
4	24.25	24.25483	5.08	– 0.00483	0.004831	123.19	123.2145	– 0.02454	0.024542
5	23.37	23.37564	7.17	– 0.00564	0.005636	167.5629	167.6033	– 0.04041	0.040408
6	22.57	22.58485	9.55	– 0.01485	0.014847	215.5435	215.6853	– 0.14179	0.141793
7	22.06	22.07157	11.35	– 0.01157	0.01157	250.381	250.5123	– 0.13132	0.131317
8	21.75	21.75871	12.54	– 0.00871	0.008712	272.745	272.8542	– 0.10925	0.109248
9	21.45	21.46152	13.73	– 0.01152	0.011516	294.5085	294.6666	– 0.15812	0.158121
10	21.09	20.988	15.73	0.101998	0.101998	331.7457	330.1413	1.604423	1.604423
11	20.68	20.69477	17.02	– 0.01477	0.014773	351.9736	352.225	– 0.25143	0.251429
12	20.22	20.23125	19.11	– 0.01125	0.011248	386.4042	386.6191	– 0.21494	0.214941
13	19.76	19.77119	21.2	– 0.01119	0.011193	418.912	419.1493	– 0.2373	0.237296
14	19.36	19.36625	23	– 0.00625	0.006252	445.28	445.4238	– 0.14379	0.143786
15	18.86	18.86664	25.08	– 0.00664	0.00664	473.0088	473.1753	– 0.16652	0.166523
16	18.27	18.27478	27.17	– 0.00478	0.004777	496.3959	496.5257	– 0.1298	0.129803
17	17.95	17.95327	28.06	– 0.00327	0.003272	503.677	503.7688	– 0.09182	0.091822
18	17.3	17.29256	29.26	0.007436	0.007436	506.198	505.9804	0.217583	0.217583
IAE (V)				0.234845	IAE (P)		3.686110144		

**Fig. 20:**
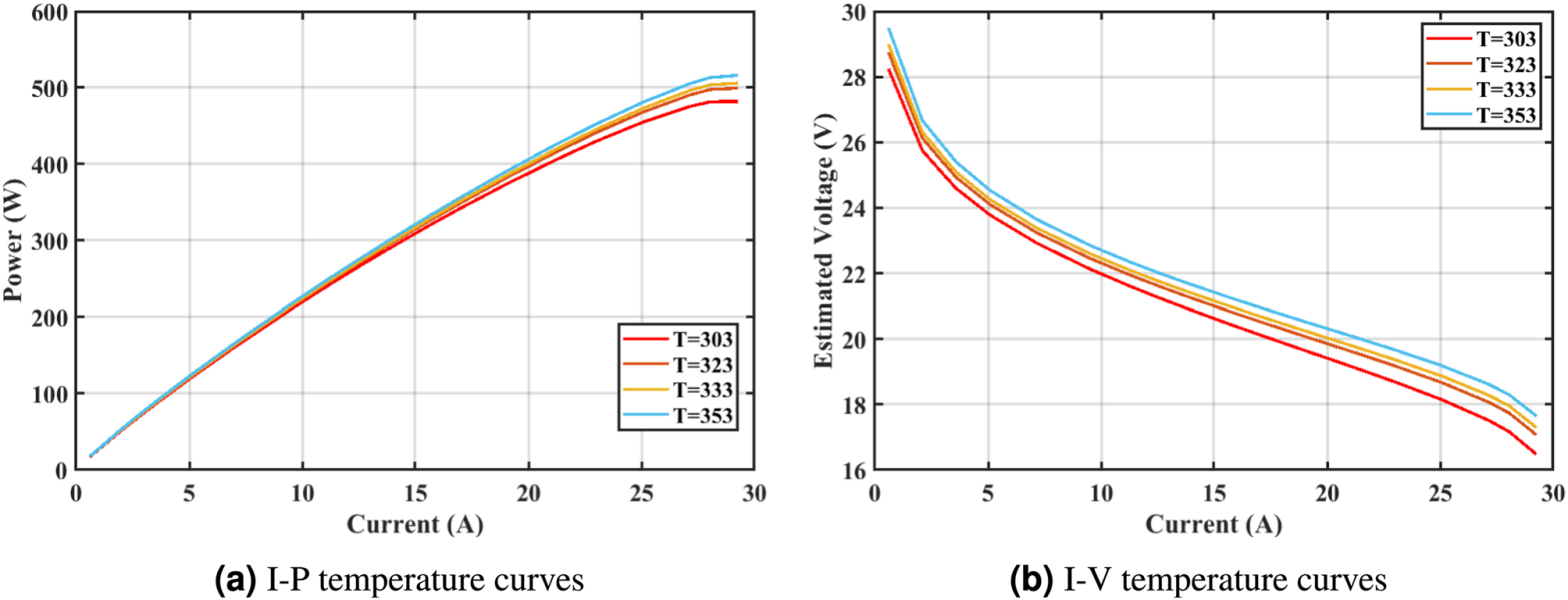
BCS 500 W stack performance plots based on GPC algorithm parameters extraction under different operating conditions.

**Fig. 21:**
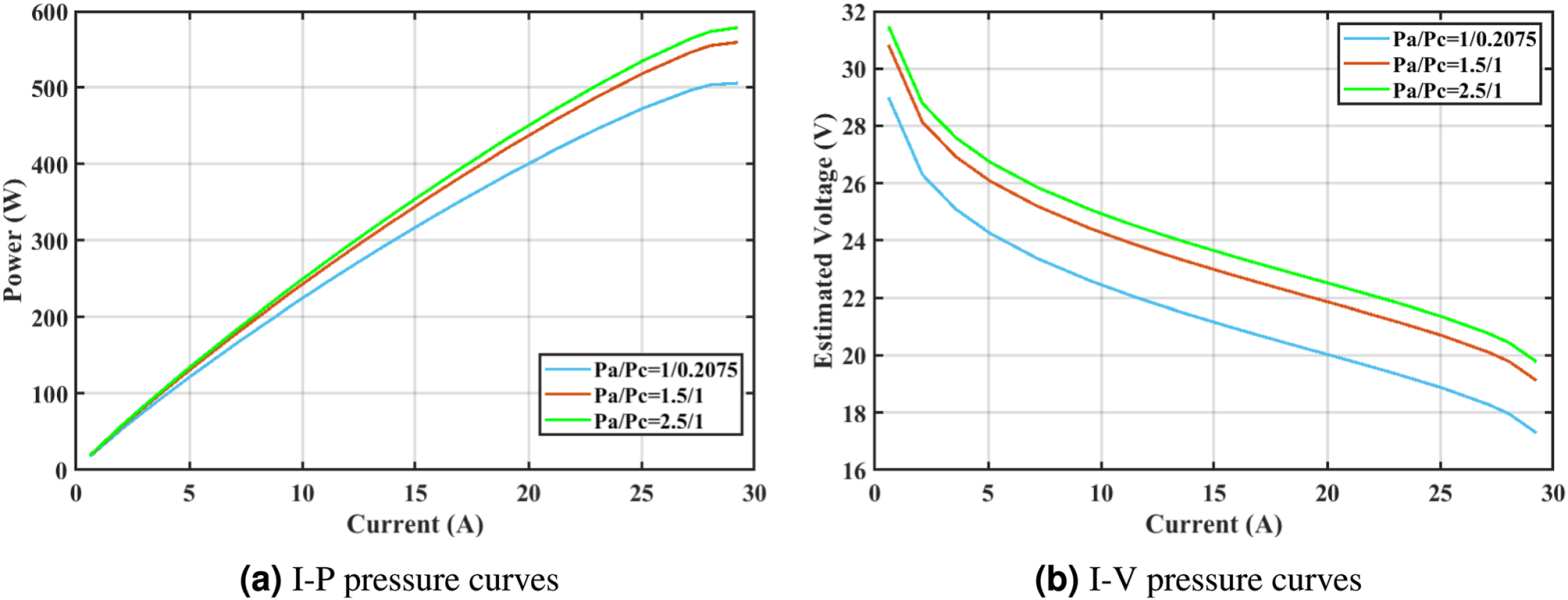
BCS 500 W stack performance plots based on GPC algorithm parameters extraction under different operating conditions.

Furthermore, the simulation results of the GPC algorithm-based PEMFC model have been obtained in varying temperature and pressure scenarios. Figure 20a and b display the I-P and I-V characteristics of this PEMFC model at different temperatures (303, 323, 333, and 353 K). The pressures $P_{H2}$/ $P_{O2}$ have been kept constant (1.000/0.2075 (bar)). It is clear that the voltage as well as the power of the PEMFC increase as the temperature of the PEMFC increases. Furthermore, Fig. 21a and b show the PI and PI characteristics of this PEMFC model at different pressures (1.000 / 0.21075, 1.5 / 1, and 2.5 / 1.000 bar) and maintained constant temperature (333K). It is significant to note that an increase in the $P_{H2}$ / $P_{O2}$, results in an increase in the voltage and power output of the PEMFC. As a result, these pressures can be precisely adjusted to achieve the desired output power from the PEMFC according to particular environmental conditions. Friedman and Wilcoxon rank tests of various MH algorithms for BCS500-W PEMFC are given in Table 16. From Table 16 and box plots in Fig. 23, it is clearly seen that the GPC algorithm obtained the lowest Friedman rank (1.970593), and based on the Friedman rank, the GPC algorithm achieved $\hbox {I}^{st}$ rank. The Wilcoxon ranking test results indicate that GPC significantly outperforms the others, evidenced by 465 winner, no losses, and minimal p-values ranging from 3.02E–11 to 4.50E–11. The Friedman and Wilcoxon ranking test clearly demonstrates that the GPC algorithm is superior in terms of precision and accuracy compared to the MH algorithms.

**Table 15:** Parameter estimation and statistical measures comparison of various MH algorithms for BCS 500 W PEMFC.

S.no	Algorithms	Objective function	$\xi _{a}$	$\xi _{b}$	$\xi _{c}$	$\xi _{d}$	$R_{con}\left( \Omega \right)$	$\beta$	$\lambda$
1	GPC	0.011699	– 0.86884	0.002854	7.64E–05	– 0.00019	0.0001	0.016136	20.88789
2	ZOA	0.012497	– 0.87852	0.002506	5.22E–05	– 0.00019	0.000251	0.015992	21.74465
3	SCHO	0.012752	– 1.11427	0.003124	4.63E–05	– 0.00019	0.000316	0.015802	22.46367
4	PSA	0.027469	– 0.8532	0.002895	8.21E–05	– 0.00019	0.0008	0.0136	24
5	SABO	0.044445	– 1.19969	0.004177	0.000098	– 0.00019	0.000171	0.016426	24
6	YDSE	0.011704	– 1.0637	0.003122	5.6E–05	– 0.00019	0.000104	0.016102	20.86594
7	EDO	0.011908	– 1.1366	0.003719	8.03E–05	– 0.00019	0.000188	0.015965	21.61097
8	RIME	0.012326	– 0.98938	0.003546	9.76E–05	– 0.00019	0.000168	0.015823	21.36505
9	CDO	1.594396	– 0.93087	0.002753	6.21E–05	– 0.00016	0.000659	0.015624	20.01874
10	COA	0.027964	– 1.19969	0.004181	0.000098	– 0.00019	0.0008	0.0136	24
11	HHO	0.028383	– 0.97184	0.003249	8.23E–05	– 0.00019	0.000372	0.015149	20.19897
12	GWO	0.01197	– 1.02867	0.002867	4.63E–05	– 0.00019	0.000112	0.016284	21.38155
13	HBA^28^	0.0118	– 0.952	0.0032	7.40E–05	– 0.000072	0.000543	0.016	20.1
14	ICA^30^	0.011856	– 0.9086	0.0024798	4.4583E–05	– 0.000193	0.000246	0.016238	22.662
15	VSDE^27^	0.01214	– 1.1970	0.0042330	9.7990E–05	– 0.000192	0.0001108	0.0157	20.194
16	SSO^90^	0.01219	– 0.8532	0.0048115	9.433E–05	– 0.000192	0.0003499	0.01589	23.000
17	AHA^22^	0.011831	– 1.0497	0.0029	3.84E–05	– 0.00019	0.00018	0.01636	22.0516
18	ShSO^24^	7.1889	– 1.018	0.0023151	5.24E–05	– 0.00012	0.000750	0.0136	18.8547
19	CS^92^	5.5625	– 1.045	0.0027788	4.59E–05	– 0.000139	0.0008	0.0136	18.4944

**Fig. 22:**
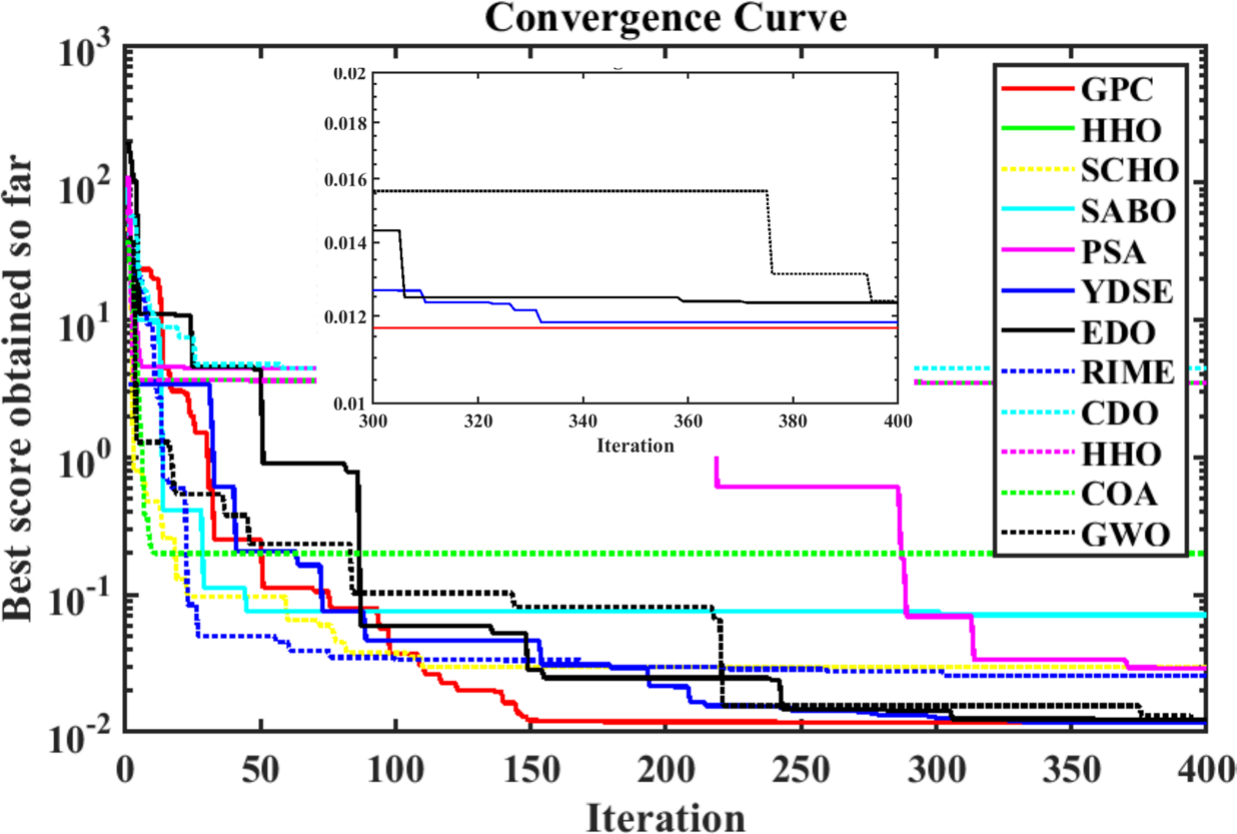
The convergence curves corresponding to BCS500-W PEMFC stack parameter extraction.

**Table 16:** Friedman and Wilcoxon rank test of various MH algorithms for BCS500-W PEMFC.

S.No	Algorithm	Friedman’s rank	RANK	Winner	Loser	Wilcoxon’s p value
1	GPC	1.970593	1			
2	ZOA	6.311832	6	465.00	0	4.5000E–11
3	SCHO	6.780215	7	465.00	0	3.0200E–11
4	PSA	9.39084	8	465.00	0	3.0200E–11
5	SABO	9.898222	9	465.00	0	3.0200E–11
6	YDSE	2.814724	2	465.00	0	3.0200E–11
7	EDO	4.472459	4	465.00	0	3.0200E–11
8	RIME	5.26032	5	465.00	0	3.0200E–11
9	CDO	12.58004	12	465.00	0	3.0200E–11
10	COA	11.03236	11	465.00	0	3.0200E–11
11	HHO	10.49754	10	465.00	0	3.0200E–11
12	GWO	4.357613	3	465.00	0	3.0200E–11

**Fig. 23:**
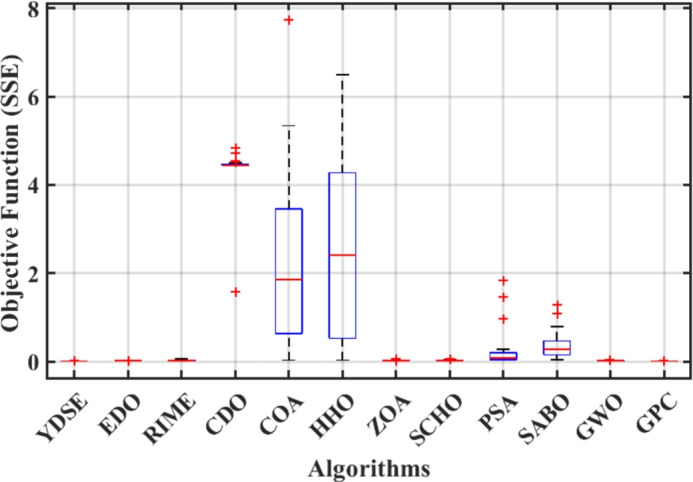
The boxplot curves obtained from 30 runs of 12 algorithms utilized to BCS500-W PEMFC stack parameter extraction.

#### Temasek Stack

**Fig. 24:**
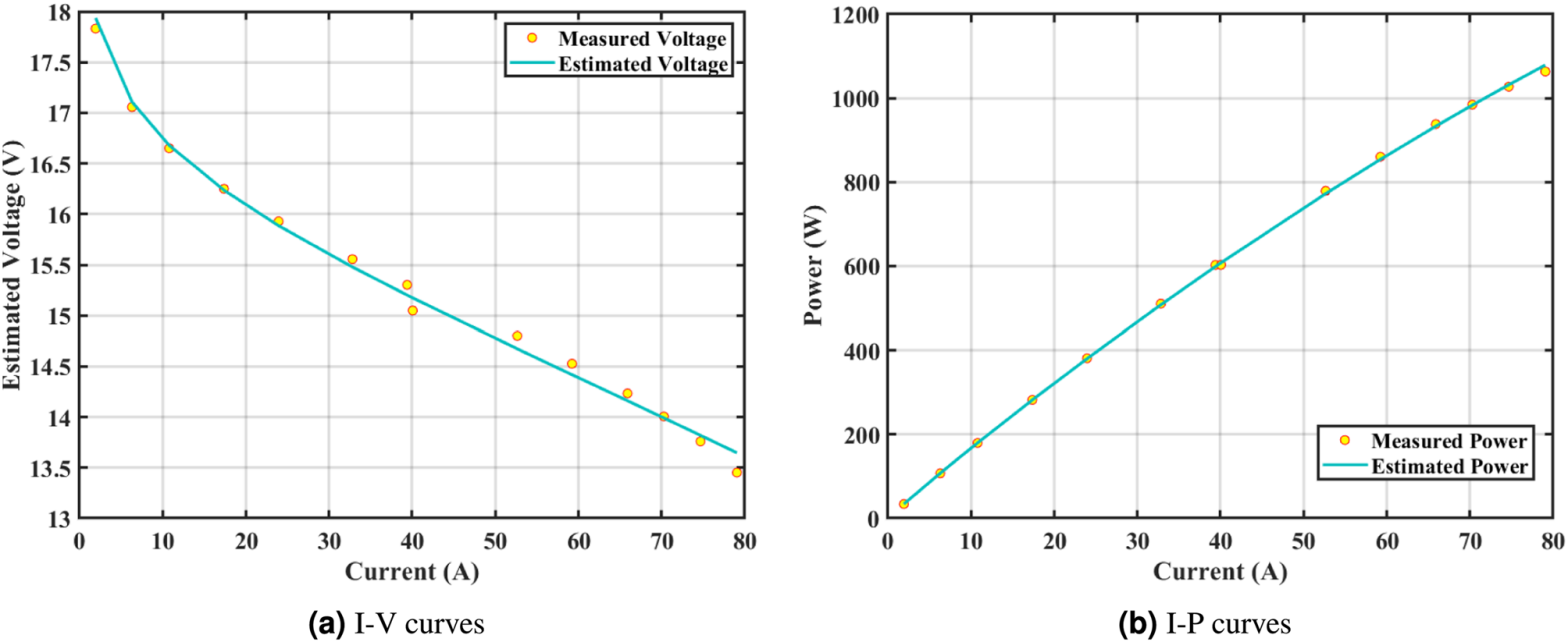
Model curves of Temasek PEMFC stack.

The Temasek Stack PEMFC stack comprises 20 individual cells that are connected in series with a membrane thickness of 51 $\mu$m^85^. The upper and lower limits and data specifications and are given in Tables 5 and 6. The experimental Power ($P_{exper}$), experimental voltage ($V_{exper}$), estimated voltage ($V_{estimat}$), model-estimated power ($P_{estimat}$), and IAE values obtained by the GPC algorithm for Temasek PEMFC are displayed in Table 17. The GPC, ZOA, SCHO, PSA, SABO, YDSE, EDO, RIME, CDO, COA, HHO, and GWO, MH optimization algorithms have been used in order to achieve optimal parameter extraction for this model. The resulting values, which have been found to be the best according to the SSE objective, have been organized and displayed in Table 18. From this table, the GPC shows superior performance along with the lowest objective function (SSE) value of 0.12327677 compared to other MH algorithms. Figure 24a and b clearly illustrate that the model curves (I-V as well as I-P curves) closely align with the experimental data for the Temasek Stack PEMFC stack and there is little variation between them. This gives further confirmation that the parameter values obtained by the proposed GPC algorithm are very precise and reliable.

**Fig. 25:**
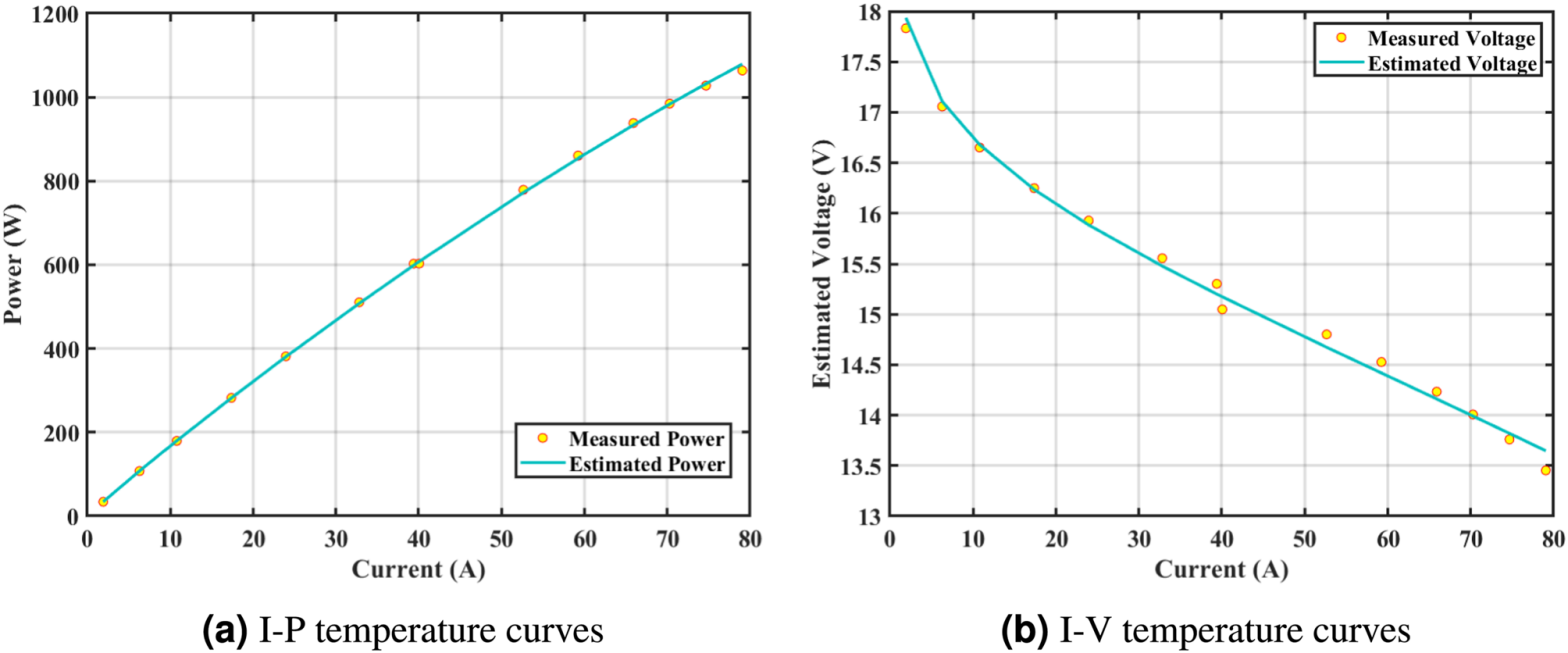
Temasek PEMFC stack performance plots based on GPC algorithm parameters extraction under different operating conditions.

**Fig. 26:**
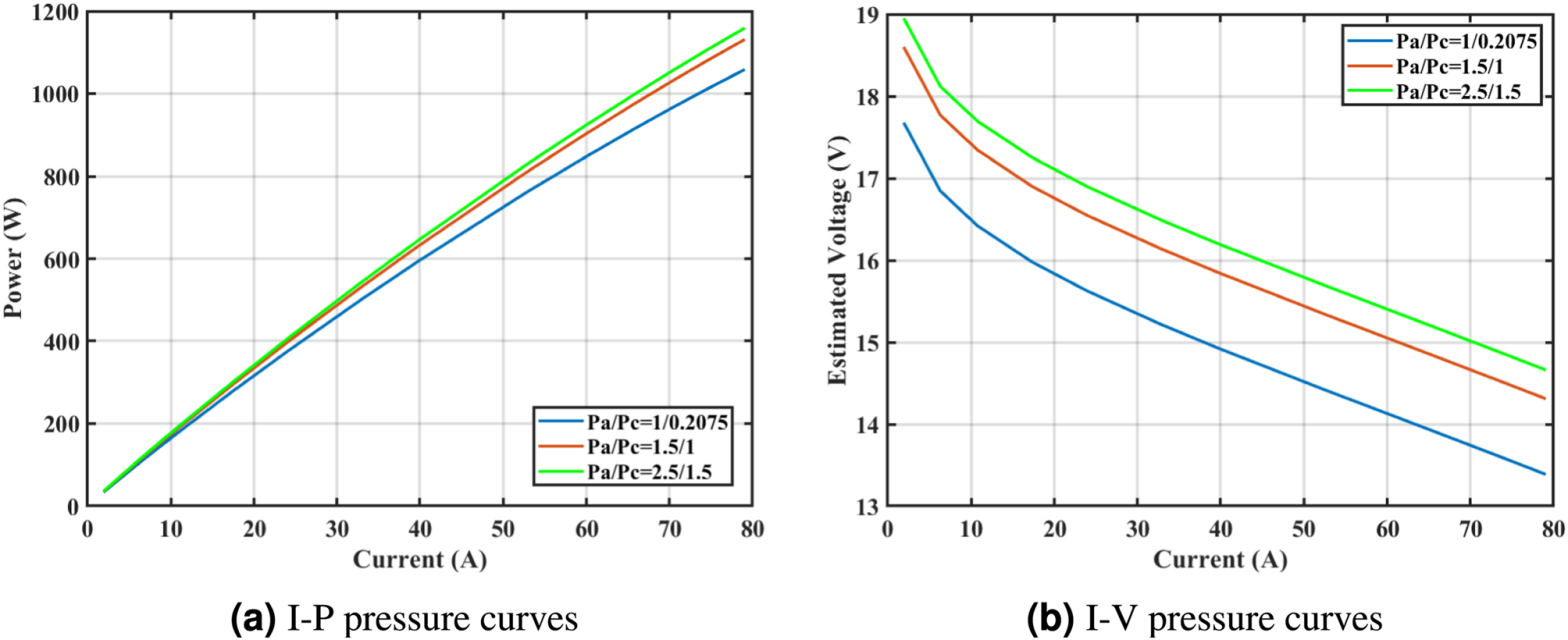
Temasek PEMFC stack performance plots based on GPC algorithm parameters extraction under different operating conditions.

**Fig. 27:**
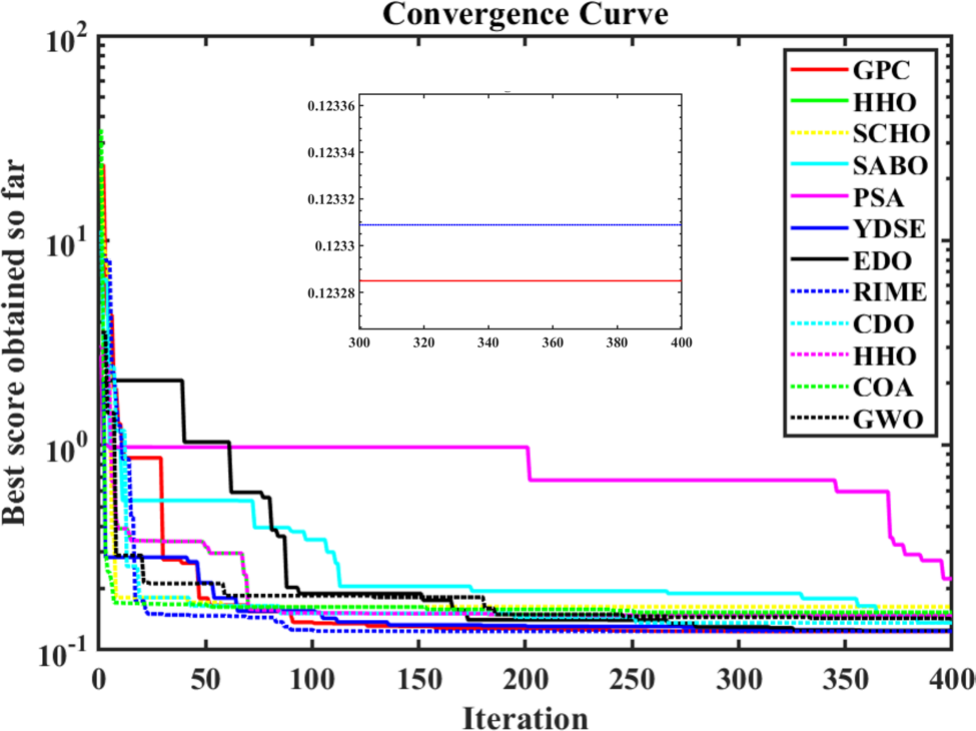
The convergence curves obtained from 400 iterations and 30 runs of 12 algorithms utilized to Temasek PEMFC stack parameter extraction.

Furthermore, temperature variations are simulated at four different temperatures: 303, 323, 333, and 353K are shown in Fig. 25a and b for I-P and I-V curves, respectively. These simulations are carried out under constant partial pressures (that is, $P_{H2}$ / $P_{O2}$ = 0.5 / 0.5 (bar)). It has been observed that as the temperature rises, there is an increase in the output voltage of the stack. Initially, the I-P and I-V curves are graphed at pressures ($P_{H2}$/ $P_{O2}$) of 1.000/0.2075, 1.5/1.000, and 2.500/1.500 bar, respectively. These measurements were taken at a constant stack temperature of 323 K and are presented in Fig. 26a and b respectively. When the supply pressures of the ($P_{H2}$/ $P_{O2}$) increase, there is an observed increase in the output voltage of the stack. Using the same simulation environment, Fig. 27, shows the convergence curves obtained from 400 iterations and 30 runs of 12 algorithms (GPC, ZOA, SCHO, PSA, SABO, YDSE, EDO, RIME, CDO, COA, HHO and GWO) used to extract the parameters of the Temasek PEMFC stack.

**Table 17:** The $V_{estimat}$, $P_{estimat}$, and IAE values obtained by the GPC algorithm for Temasek Stack PEMFC.

SNo.	$V_{exper}$	$V_{estimat}$	$I_{exper}$	$V_{exper}$- $V_{estimat}$	$\big| V_{exper}-$ $V_{estimat} \big|$	$P_{exper}$	$P_{estimat}$	$P_{exper}$- $P_{estimat}$	$\big| P_{exper}-$ $P_{estimat} \big|$
1	17.8316	17.93615	1.91584	– 0.104545056	0.104545	34.16249	34.36278	– 0.20029	0.200292
2	17.0572	17.10754	6.30236	– 0.050340604	0.050341	107.5006	107.8179	– 0.31726	0.317265
3	16.6501	16.67785	10.7842	– 0.027747647	0.027748	179.558	179.8572	– 0.29924	0.299236
4	16.2495	16.23595	17.364	0.01355424	0.013554	282.1563	281.921	0.235356	0.235356
5	15.9291	15.88514	23.9438	0.043958036	0.043958	381.4032	380.3507	1.052522	1.052522
6	15.5553	15.4775	32.8122	0.077798612	0.077799	510.4036	507.8509	2.552744	2.552744
7	15.3016	15.20048	39.392	0.101116044	0.101116	602.7606	598.7775	3.983163	3.983163
8	15.048	15.17284	40.0672	– 0.124835175	0.124835	602.9312	607.933	– 5.0018	5.001796
9	14.801	14.67338	52.6469	0.127616904	0.127617	779.2268	772.5081	6.718634	6.718634
10	14.5273	14.41849	59.2267	0.108814114	0.108814	860.404	853.9593	6.444701	6.444701
11	14.2336	14.16067	65.9019	0.072925389	0.072925	938.0213	933.2154	4.805922	4.805922
12	14.0066	13.99067	70.2884	0.015931862	0.015932	984.5015	983.3817	1.119825	1.119825
13	13.7597	13.81959	74.6749	– 0.059888779	0.059889	1027.504	1031.976	– 4.47219	4.472189
14	13.4526	13.64697	79.0614	– 0.194373056	0.194373	1063.581	1078.949	– 15.3674	15.36741
IAE (V)					1.123446	IAE (P)			52.57105

**Table 18:** Parameter estimation and statistical measures comparison of various MH algorithms for Temasek Stack PEMFC.

S.no	Algorithms	Objective function	$\xi _{a}$	$\xi _{b}$	$\xi _{c}$	$\xi _{d}$	$R_{con}\left( \Omega \right)$	$\beta$	$\lambda$
1	GPC	0.123276775	– 0.9927	0.003072	5.81E–05	– 9.54E–05	0.0001	0.101876	10
2	ZOA	0.12328198	– 0.86374	0.002357	3.65E–05	– 9.54E–05	0.0001	0.102076	10.00082
3	SCHO	0.124342719	– 1.01632	0.003146	5.82E–05	– 9.55E–05	0.000109	0.100392	10
4	PSA	0.123312657	– 0.8532	0.002317	3.60E–05	– 9.54E–05	0.0001	0.10238	10
5	SABO	0.127516141	– 0.8532	0.002316	3.60E–05	– 9.54E–05	0.000143	0.095196	10
6	YDSE	0.123291279	– 1.03225	0.003767	9.74E–05	– 9.54E–05	0.0001	0.101588	10
7	EDO	0.123691394	– 1.0783	0.003787	8.90E–05	– 9.54E–05	0.0001	0.103136	10
8	RIME	0.123276797	– 1.09176	0.003055	3.60E–05	– 9.54E–05	0.0001	0.101864	10
9	CDO	0.124344819	– 0.8532	0.002337	3.73E–05	– 9.54E–05	0.0001	0.104545	10
10	COA	0.123310904	– 0.8532	0.002317	3.60E–05	– 9.54E–05	0.0001	0.102109	10
11	HHO	0.127156269	– 0.8532	0.002383	4.05E–05	– 9.54E–05	0.000113	0.115089	11.25314
12	GWO	0.124192754	– 0.91252	0.002968	6.80E–05	– 9.54E–05	0.000117	0.099262	10
13	SSO^24^	1.6481	– 1.0299	0.0024105	4.00E–05	– 9.54E–05	0.0001087	0.1274	10.0005
14	FPA^31^	0.1881	– 0.4838	0.001	2.7739E–05	– 7.57E–05	0.0001109	0.1287	11.3223

**Fig. 28:**
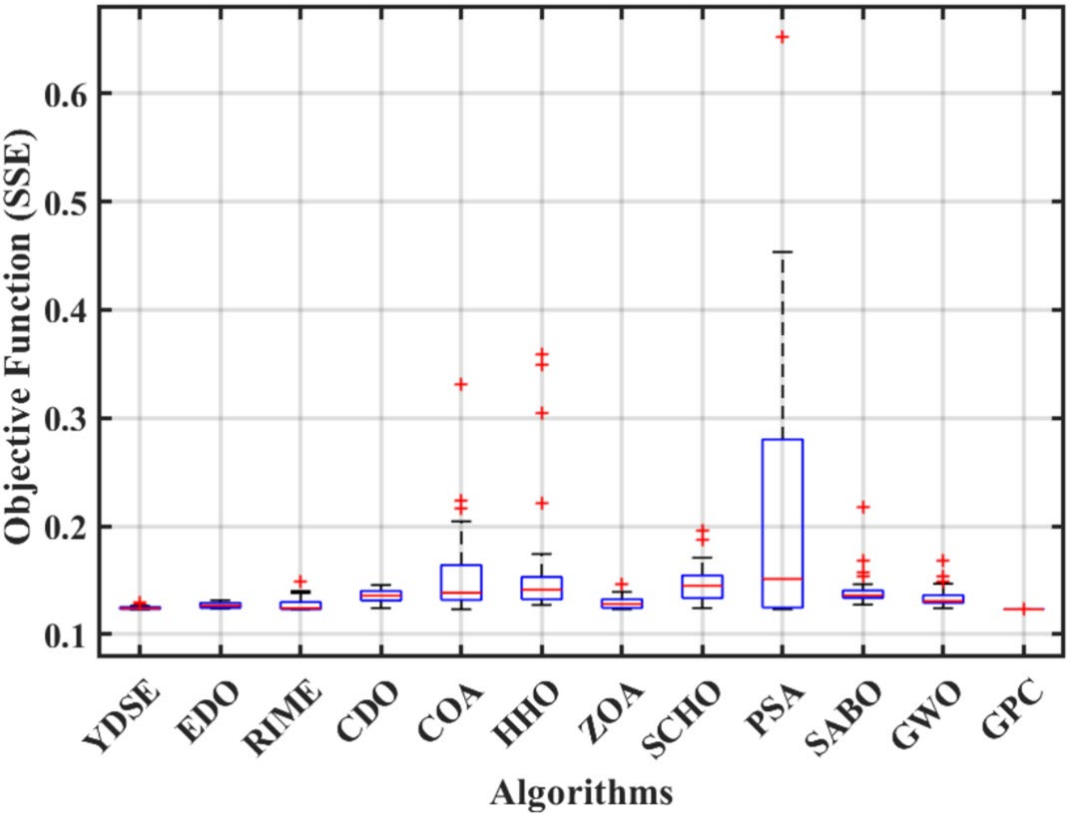
The boxplot curves obtained from 30 runs of 12 algorithms utilized to Temasek PEMFC stack parameter extraction.

Figure 28 shows the box plot curves obtained from 30 runs of the GPC, ZOA, SCHO, PSA, SABO, YDSE, EDO, RIME, CDO, COA, HHO and GWO algorithms used to extract the parameters from the Temasek PEMFC stack. Based on the size of the box and the number of outliers, it is clear from Fig. 28 that the proposed GPC algorithm outperforms the other 11 algorithms. Table 19 presents the Friedman and Wilcoxon rank test of various MH algorithms for the Temasek PEMFC stack. From Table 16 it is clearly seen that the GPC algorithm obtained the lowest Friedman rank (2.503926), and based on the Friedman rank, the GPC algorithm achieved $\hbox {I}^{st}$ rank. The Wilcoxon ranking test results indicate that GPC significantly outperforms the others, evidenced by 465 winner, no losses, and minimal p-values < 0.05 in all cases. The Friedman and Wilcoxon ranking test clearly demonstrates that the GPC algorithm is superior in terms of precision and accuracy compared to the MH algorithms.

**Table 19:** Friedman and Wilcoxon rank test of various MH algorithms for Temasek PEMFC.

S.No	Algo	Friedman’s rank	Rank	Winner	Loser	Wilcoxon’s p value
1	GPC	2.503926	1	−	−	$-$
2	GPC	5.611832	4	465	0	6.01E–08
3	SCHO	9.846882	11	465	0	3.02E–11
4	PSA	10.22417	12	465	0	2.87E–10
5	SABO	9.231555	10	465	0	3.02E–11
6	YDSE	4.18139	2	465	0	1.01E–08
7	EDO	5.772459	5	465	0	3.02E–11
8	RIME	4.293653	3	465	0	0.000201
9	CDO	8.213376	7	465	0	3.02E–11
10	COA	9.032359	8	465	0	1.33E–10
11	HHO	9.164207	9	465	0	3.02E–11
12	GWO	7.290946	6	465	0	3.02E–11

## Conclusions

This paper presents a novel multi-hybrid optimization algorithm, known as the hybrid Gray Particle Cuckoo (GPC) algorithm, to identify unknown parameters of the PEMFC stack. Determining the values of the unknown model parameters ($\xi _a$, $\xi _b$, $\xi _c$, $\xi _d$, $R_{con}$, $\lambda$, and $\beta$) is a crucial subject in the discipline of PEMFC research. However, the complex nature of the PEMFC system makes it a very difficult challenge. Four different commercial PEMFCs (BCS500-W, Ballard Mark V, NedStack PS6, and Temasek Stack) were examined to determine their unknown parameters utilizing the GPC algorithm. The precision of the GPC algorithm was validated by the precise correlation between the results derived from the estimated and experimentally observed results. Statistical analysis, such as SSE (minimum, mean, and std.), IAE, MBE, MAE, MSE, and RMSE has been performed to demonstrate the superiority of the GPC algorithm compared to the other 11 MH optimization algorithms (ZOA, SCHO, PSA, SABO, YDSE, EDO, RIME, CDO, COA, HHO, and GWO). The objective function has been implemented as the SSE between the estimated and experimental voltage values, and the fitness values for the four PEMFC stacks (BCS500-W, Ballard Mark V, NedStack PS6 and Temasek) are 0.011699, 0.813912, 2.267687, and 0.123276775, respectively, using the GPC algorithm. In addition, all test cases undergo a thorough evaluation of the effects of altering the input operating parameters of the PEMFCs, such as temperature and supply pressures. In addition to the PEMFC extraction challenges, the performance of the proposed algorithm has been tested using the CEC 2019 challenges, and the results achieved by the GPC algorithm have been compared with other MH optimization algorithms (FPA, BWOA, FROBLGJO, CDO, COA, HHO, ZOA, ARNMRA, YDSE, as well as jDE100) to demonstrate its superiority. Additionally, a nonparametric test analysis (Friedman and Wilcoxon signed rank test), as well as the box plot, has been performed to verify the precision and reliability of the GPC algorithm compared to existing algorithms, and it is clear that the GPC algorithm is superior.

The performance variability of GPC in different benchmarks and PEMFC datasets can be attributed to the interaction between the internal dynamics of the algorithm and the inherent characteristics of the datasets themselves. In cases where the landscape of the underlying parameters is highly multimodal, with numerous local optima, GPC tends to outperform standalone algorithms due to its phase-wise integration of exploration and exploitation strategies. Early stage Lévy flights from CS help escape deceptive basins, while the guided search of GWO and the convergence strength of PSO allow for effective refinement. This layered adaptability is particularly effective in data sets with non-linear interdependencies and irregular error surfaces. However, in scenarios where the optimization landscape is relatively smooth or low-dimensional, simpler algorithms with fewer control parameters, such as standard PSO or GWO, may yield comparable or better results due to their lower overhead and faster convergence. Thus, the advantage of GPC becomes more pronounced in complex, noisy, or ill-conditioned datasets, while its performance may converge to baseline methods in well-behaved or low-complexity data sets. This suggests a potential avenue for future work, adapting the degree of hybridization dynamically based on landscape analysis or preliminary fitness landscape sampling.

Future studies should prioritize the validation of the proposed GPC algorithm for different fuel cell technologies, solar photovoltaic parameter extraction, smart grids, and other real-world applications. The GPC algorithm can also be improved for better solution quality by using new equations for exploration and exploitation operations. Other important factors can be the introduction of population size reduction, a memory bank to store previous solutions, and the reduction of computational time for better performance of the proposed GPC algorithm.

## Data Availability

The datasets used and/or analysed during the current study available from the corresponding author on reasonable request. Funding Statement: We gratefully acknowledge the funding support by program “Excellence initiative—research university” for the AGH University of Kraków, Poland as well as the ARTIQ project: UMO-2021/01/2/ST6/00004 and ARTIQ/0004/2021.

## Abbreviations

FC: Fuel Cell
PEMFC: Proton Exchange Memberane Fuel Cells
GWO: Grey wolf optimizer
PSO: Particle Swarm Optimization
CS: Cuckoo Search
SABO: Subtraction Average based Optimizer
YDSE: Young Double Slit Experiment
EDO: Exponential Distribution Optimizer
BWOA: Black Widow Optimization Algorithm
FROBLGJO: Fast Random Opposition-based Learning Golden jackal optimization
jDE100: Self-adaptive DE
ARNMRA: Attraction and Repulsion-based Naked Mole Rat Algorithm
IHBO: Improved Heap-based Optimizer
LSA: Lightning Search Algorithm
STLBO: Simplified Teaching Learning Based Optimization
AHA: Artificial Hummingbird Algorithm
RGWO: Repairable Grey Wolf Optimization
ShSO: Shark Smell Optimize
MRFO: Manta Rays Foraging Optimizer
VSDE: Hybrid Vortex Search Differential Evolution
HBOA: Honey Badger Optimization Algorithm
IABC: Improved Artificial Bee Colony
ICA: Imperialist Competitive Algorithm
FPA: Flower Pollination Algorithm
TSA: Transient Search Optimization
DO: Dandelion Optimize
IFMO: Improved Fish Migration Optimizer
ABC-DE: Artificial Bee Colony Differential Evolution
CBO: Chaotically based bonobo optimize
FOA: Firefly Optimization Algorithm
SMBOA: Slime Mould based Optimization Algorithm
ESSA: Enhanced Salp Swarm Algorithm
HBO: Honey Badger Optimizer
AGPSO: Autonomous Groups Particle Swarm Optimization
GTO: Gorilla Troops Optimizer
EWO: Enhanced Walrus Optimization
SSO: Salp Swarm Optimizer
IAEO: Improved Artificial Ecosystem Optimizer
OOA: Osprey Optimization Algorithm
NNO: Neural Network Optimizer
WSO: War Strategy Optimization
IFMO: Improved Fish Migration Optimizer
ZOA: Zebra Optimization Algorithm
PSA: Propagation Search Algorithm
CDO: Chernobyl Disaster Optimizer
COA: Coati Optimization Algorithm
NFLT: No Free-Lunch Theorem
MH: Meta-heuristic
CEC: Congress on Evolutionary Computation
MAE: Mean Absolute Error
IAE: Individual Absolute Error
MBE: Mean Bias Error
MSE: Mean Square Error
RMSE: Root-Mean Square Error
TSEs: Total Squared Errors
SQEs: Sum of Quadratic Errors
SSE: Sum of Square Error
